# Targeting JUN, CEBPB, and HDAC3: A Novel Strategy to Overcome Drug Resistance in Hypoxic Glioblastoma

**DOI:** 10.3389/fonc.2019.00033

**Published:** 2019-02-01

**Authors:** Yixing Gao, Bao Liu, Lan Feng, Binda Sun, Shu He, Yidong Yang, Gang Wu, Guoji E, Chang Liu, Yuqi Gao, Erlong Zhang, Bo Zhu

**Affiliations:** ^1^Department of Oncology, Xinqiao Hospital, Army Medical University, Chongqing, China; ^2^Institute of Medicine and Equipment for High Altitude Region, College of High Altitude Military Medicine, Army Medical University, Chongqing, China; ^3^Key Laboratory of High Altitude Medicine, People's Liberation Army, Chongqing, China

**Keywords:** glioblastoma, hypoxia, drug resistance, JUN, CEBPB, HDAC3

## Abstract

Hypoxia is a predominant feature in glioblastoma (GBM) and contributes greatly to its drug resistance. However, the molecular mechanisms which are responsible for the development of the resistant phenotype of GBM under hypoxic conditions remain unclear. To analyze the key pathways promoting therapy resistance in hypoxic GBM, we utilized the U87-MG cell line as a human GBM cell model and the human brain HEB cell line as a non-neoplastic brain cell model. These cell lines were cultured in the presence of 21, 5, and 1% O_2_ for 24 h. We detected the changes in transcriptional profiling and analyzed the biological processes and functional interactions for the genes with different expression levels under different hypoxia conditions. The results indicated that those alterations of U87-MG cells presented specific transcriptional signature in response to diverse hypoxia levels. Gene ontology analysis revealed that the genes related to the DNA replication and cell cycle were suppressed, while the genes involved in tissue and system development to promote cancer development were activated following hypoxia. Moreover, functional interaction analysis suggested that the epigenetic regulator HDAC3 and the transcriptional factors CEBPB and JUN played a central role in organ and system developmental process pathway. Previous studies reported the global alterations caused by activation of HDAC3, CEBPB, and JUN could form the molecular basis of the resistance to chemotherapy and radiation therapy of hypoxic GBM. In our study, the significant growth inhibitory effect of temozolomide on hypoxic GBM cells could be promoted under downregulation of these genes. The experiment suggested that HDAC3, CEBPB, and JUN were closely involved in the drug-resistance phenotype of hypoxic GBM. In summary, we profiled the hypoxia-dependent changes in the transcriptome of the U87-MG cell line and the human brain cell line HEB to identify the transcriptional signatures of U87-MG cells and elucidate the role of hypoxia in the drug-resistant phenotype of GBM. Furthermore, we identified three key genes and explored their important roles in the drug resistance of hypoxic GBM.

## Introduction

Glioblastoma (GBM) is the most common malignant and aggressive primary brain tumor in humans (1). Despite current multimodality treatment efforts that include possible maximal surgical resection and combination of radiotherapy and/or chemotherapy, the median survival is estimated to 15 months (2). The poor prognosis is most often ascribed to the resistance of GBM to chemotherapeutic agents and radiotherapy as well as to its inherent complexities at the molecular level (3). The reason of chemotherapy failure contains three aspects, namely inadequate drug pharmacokinetic properties, intrinsic factors (the expression of drug efflux pumps) and tumor microenvironment-related factors (4, 5). The resistance mechanisms of the intrinsic factors are mediated by genes associated with drug efflux and DNA repair (6, 7) including ATP binding cassette subfamily B member 1 (ABCB1), the DNA repair protein O^6^-methylguanine-DNA methyltransferase (MGMT), the DNA mismatch repair and the DNA base excision repair systems (8, 9).

Recently, it was shown that the tumor microenvironment, which was characterized by hypoxic regions promoted resistance of solid tumors to current therapy (10). Due to the rapid proliferation of tumor cells and insufficient supply of blood vessels, a microenvironment is formed that is characterized hypoxic regions. Thus, hypoxia had been regarded as a predominant feature for GBM (11). Liang (12) reported that hypoxic exposure could significantly increase the drug resistance of glioma cells. However, this property indicated no correlation with the expression of the multidrug resistance genes (MDR1, MRP, 06MT, and ERCC), which implied that other mechanisms might be acting in these hypoxic tumors (12). Subsequently, several studies supported that the HIF family of hypoxia-inducible transcription factors represented the main mediator of the hypoxic response of tumor cells. HIFs could promote drug resistance by regulating drug efflux, altering cell proliferation and survival, inhibiting DNA damage, mitochondrial activity and metabolic reprogramming, and modifying stromal cell morphology and autophagy (4, 13–16). However, the therapeutic strategies targeting HIF-1α are not able to eradicate tumors selectively (17), since HIFs participate in several physiological activities (18). This suggests that a novel mechanism should be explored that defines the GBM drug-resistance phenotype under hypoxic conditions. The comprehensive understanding of the response of GBM to hypoxia will aid the identification of the efficient agents for GBM treatment.

With the progression of sequencing technology, the genome-wide profiling of several cancer types provided deep insights in the molecular basis of tumor initiation and progression. Recently, genome-wide transcriptome analysis in human glioma cells has revealed specialized gene signatures related to the response of GBM to ionizing radiation and to the development of the corresponding resistance to this type of treatment (through activation of anti-apoptotic genes) (19). In the present study, we performed deep RNA sequencing to clarify the functions of GBM cells under hypoxia. Furthermore, by functional interaction analysis we identified the key genes and investigated their role in the drug resistance of hypoxic GBM cells. The data indicated that three genes, namely JUN, CEPBP, and HDAC3 were mainly involved in the drug resistance of hypoxic GBM cells and could be regarded as potential therapeutic targets for this disease.

## Results

### Biological Activities of Human GBM Cells Under Hypoxia

**Figure 1:**
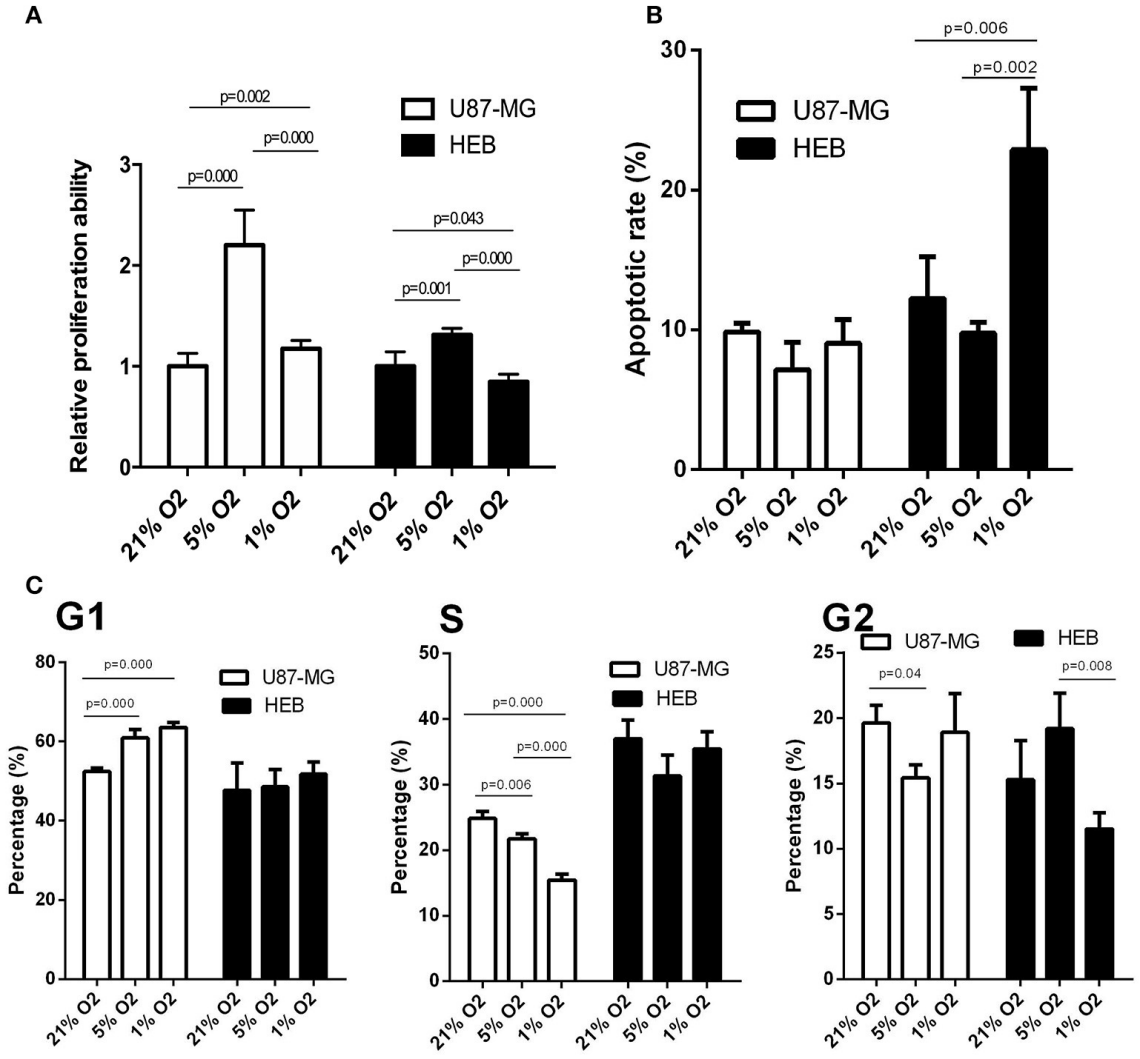
Effects of hypoxia on cell proliferation, apoptosis and cell cycle in U87-MG and HEB cells. U87-MG and HEB cells were cultured in the presence of 21, 5, and 1% O_2_ for 24 h. **(A)** Cell proliferation was evaluated by the CCK-8 assay. **(B)** Cell apoptosis was measured by flow cytometric analysis of cells labeled with Annexin V/PI double staining. **(C)** The cell cycle was examined by flow cytometry. All experiments were independently repeated three times. All data are presented as mean ± SD.

U87-MG and HEB cells were cultured in the presence of 21, 5, and 1% O_2_ for 24 h, and their proliferation was assessed by the survival assay. The cell proliferation rates of U87-MG and HEB cells were significantly increased in the presence of 5%O_2_, whereas in the presence of 1% O_2_, only U87-MG cells presented significantly increased proliferation and the proliferation of HEB cells was decreased obviously (Figure 1A). Furthermore, hypoxia did not induce apoptosis of U87-MG cells. In contrast to these findings, HEB cells presented a higher apoptotic rate in the presence of 1% O_2_ compared with that noted under normoxic conditions (Figure 1B). These results revealed that 1% O_2_ hypoxia could inhibit proliferation and promote apoptosis of normal cells as opposed to GBM cells. Moreover, the results of the flow cytometry assays indicated that treatment of U87-MG cells with 1% O_2_ increased the proportions of the cells at the G1 phase and decreased the percentage of the cells at the S phase compared with the corresponding percentages of the cells cultured in the presence of 21% O_2_ (Figure 1C), which indicated that hypoxia could induce G1 arrest of GBM cells.

### Global Changes in Gene Expression in Response to Hypoxia

**Figure 2:**
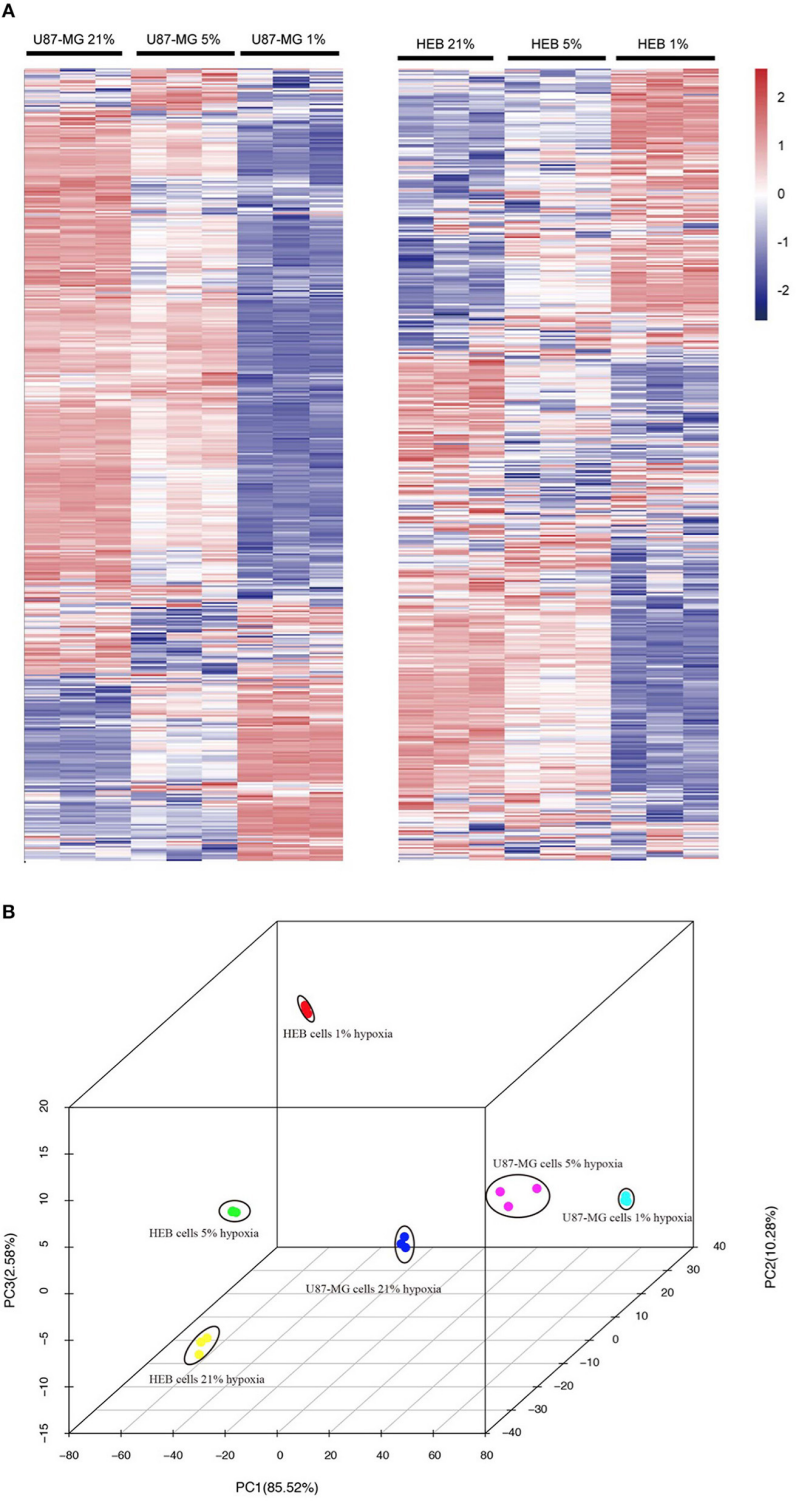
Gene expression profiles of U87-MG and HEB cells in response to hypoxia. The samples from U87-MG and HEB cells were cultured in the presence of 21, 5, and 1% O_2_ for 24 h. **(A)** All analyzed genes (except FPKM < 1) were subjected to K-mean clustering. The genes that exhibited abundance above the mean were shown in red, whereas those that were below and/or equivalent to the mean were depicted in blue, white, respectively. **(B)** Principle component plot of the genes under the different hypoxic levels of U87-MG and HEB cell incubation. All the data were analyzed from three individual tests.

Unsupervised hierarchal clustering and PCA were used to visualize the overall response of the gene expression to the graded levels of hypoxia. The cells could form distinct clusters under different hypoxic conditions (Figures 2A,B). Moreover, both U87-MG and HEB cells presented considerable changes in gene expression profiling under 1% O_2_ conditions.

**Figure 3:**
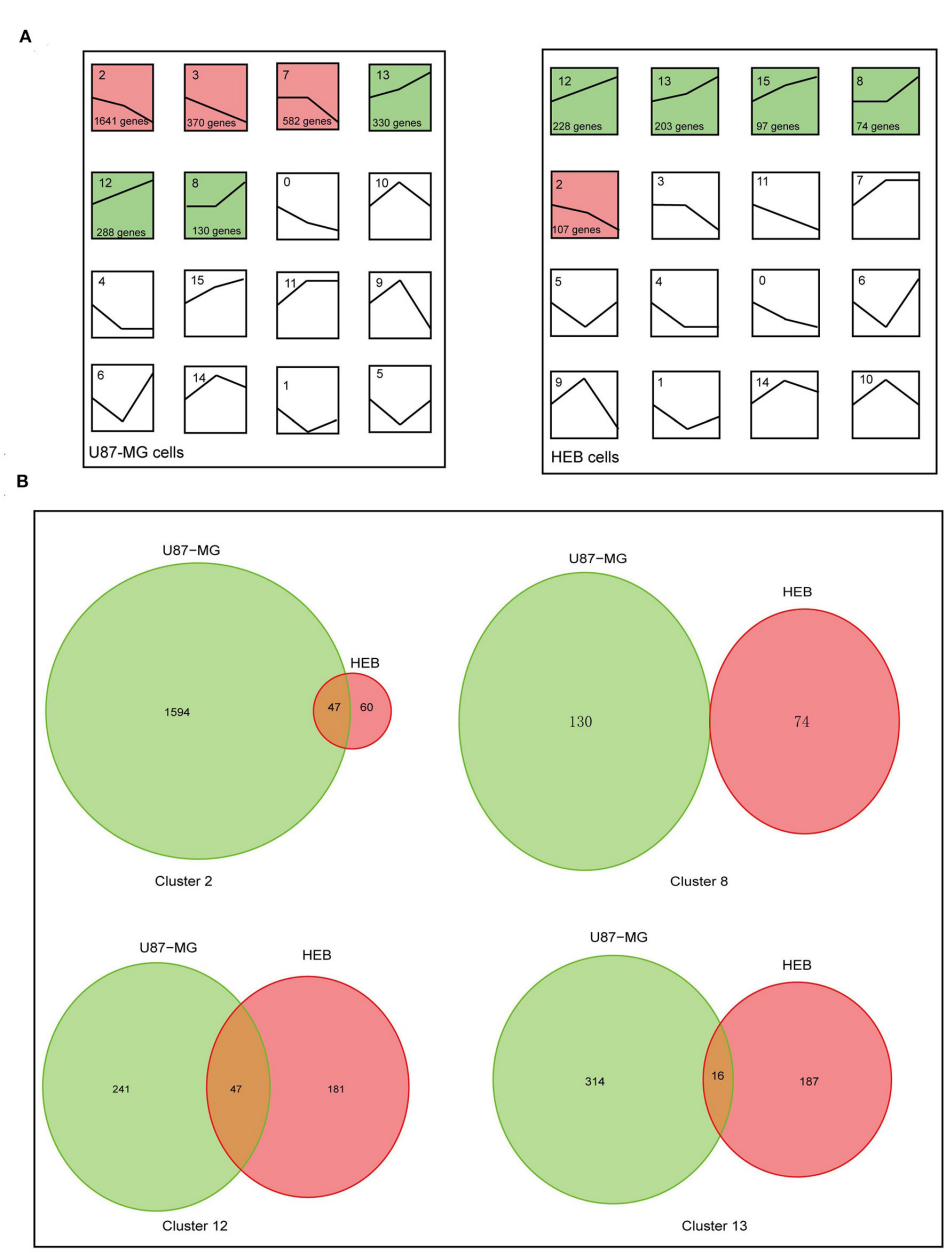
Changes of gene expression levels in U87-MG and HEB cells in the presence of different levels of hypoxia. **(A)** Significant changes of gene expression in U87-MG and HEB cells. The global expression profiles of U87-MG were clustered in 6 clusters, including 3 upregulated patterns (cluster 8, 12, and 13) and 3 downregulated patterns (cluster 2, 3, and 7), while HEB cells were clustered in 5 clusters, containing 4 upregulated patterns (cluster 8, 12, 13, and 15), and 1 downregulated pattern (cluster 2). For each cluster the number of genes assigned was presented at the lower left corner of the cluster box. **(B)** Venn diagrams indicated overlap of hypoxia-induced genes under the different hypoxic conditions of U87-MG and HEB cell incubation. The clusters 2, 8, 12, and 13 were common in U87-MG and HEB cells. All the data were from three individual tests.

To examine the global gene expression profiles of U87-MG and HEB cells, the expression data (from v21 to v1% O_2_) were normalized to 0, log_2_ (v5/v21%) and log_2_ (v1/v21%). The global gene expression profiles of U87-MG were clustered in 6 clusters, including 3 upregulated patterns (cluster 8, 12, and 13) and 3 downregulated patterns (cluster 2, 3, and 7). HEB cells were clustered in 5 clusters, containing 4 upregulated patterns (cluster 8, 12, 13, and 15) and 1 downregulated pattern (cluster 2) (*p* ≤ 0.05; Figure 3A). The clusters 2, 8, 12, and 13 were shared in U87-MG and HEB cells. However, the genes identified in the 4 clusters were considerably different between U87-MG and HEB cells. The number of common genes in clusters 2, 8, 12, and 13 were 47 (2.8%), 0 (0%), 47 (10%), and 16 (3.1%), respectively (Figure 3B).

### Biological Processes Responses Induced by Hypoxia

**Figure 4:**
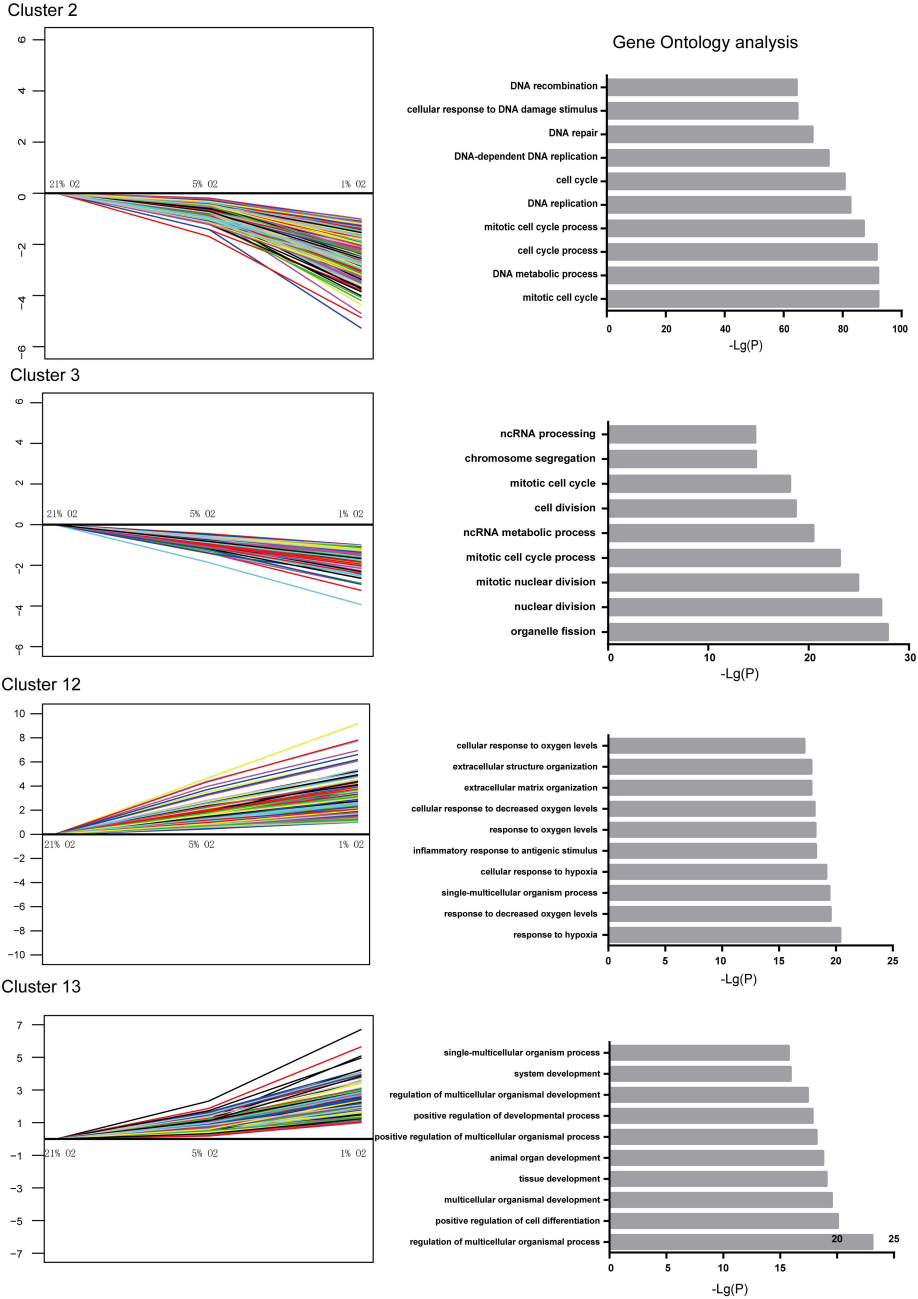
Significantly altered gene expression profiles and their GO classification in U87-MG cells. Clusters 2 and 3 indicated a downregulated trend, whereas clusters 12 and 13 indicated an upregulated trend following incubation of the cells in the presence of 21–1% O_2_. All the data were from three individual tests.

The genes within the up- and downregulated cluster groups were subjected to gene ontology (GO) analysis. In U87-MG cells, cluster 2 and 3 genes were the most enriched genes involved in DNA replication, cell cycle and cell division, indicating a mechanism of hypoxia-induced cell growth arrest. The most enriched genes found in cluster 12 were those that were involved in the response to hypoxia and the inflammatory response to antigenic stimuli. It is interesting to note that various genes involved in the positive regulation of cell differentiation, tissue development and system development were found in cluster 13 (Figure 4). The genes identified in clusters 7 and 8 did not present any significant difference in their GO terms.

Taken together, these observations indicated marked alterations in the biological processes of U87-MG cells following hypoxic stimuli. The genes involved in the cell cycle and DNA replication were suppressed by hypoxia, whereas the genes that were involved in the response to hypoxia, positive regulation of cell differentiation, tissue development and system development were activated under hypoxic conditions. Moreover, the expression levels of the genes associated with positive regulation of cell differentiation, tissue development and system development exhibited significant changes following incubation of the cells in the presence of 1% O_2_ compared with the levels noted in the presence of 5 and 21% O_2_ treatment.

### JUN, CEBPB, and HDAC3 Played a Key Role in the Regulation of the Genes Under Hypoxia

**Figure 5:**
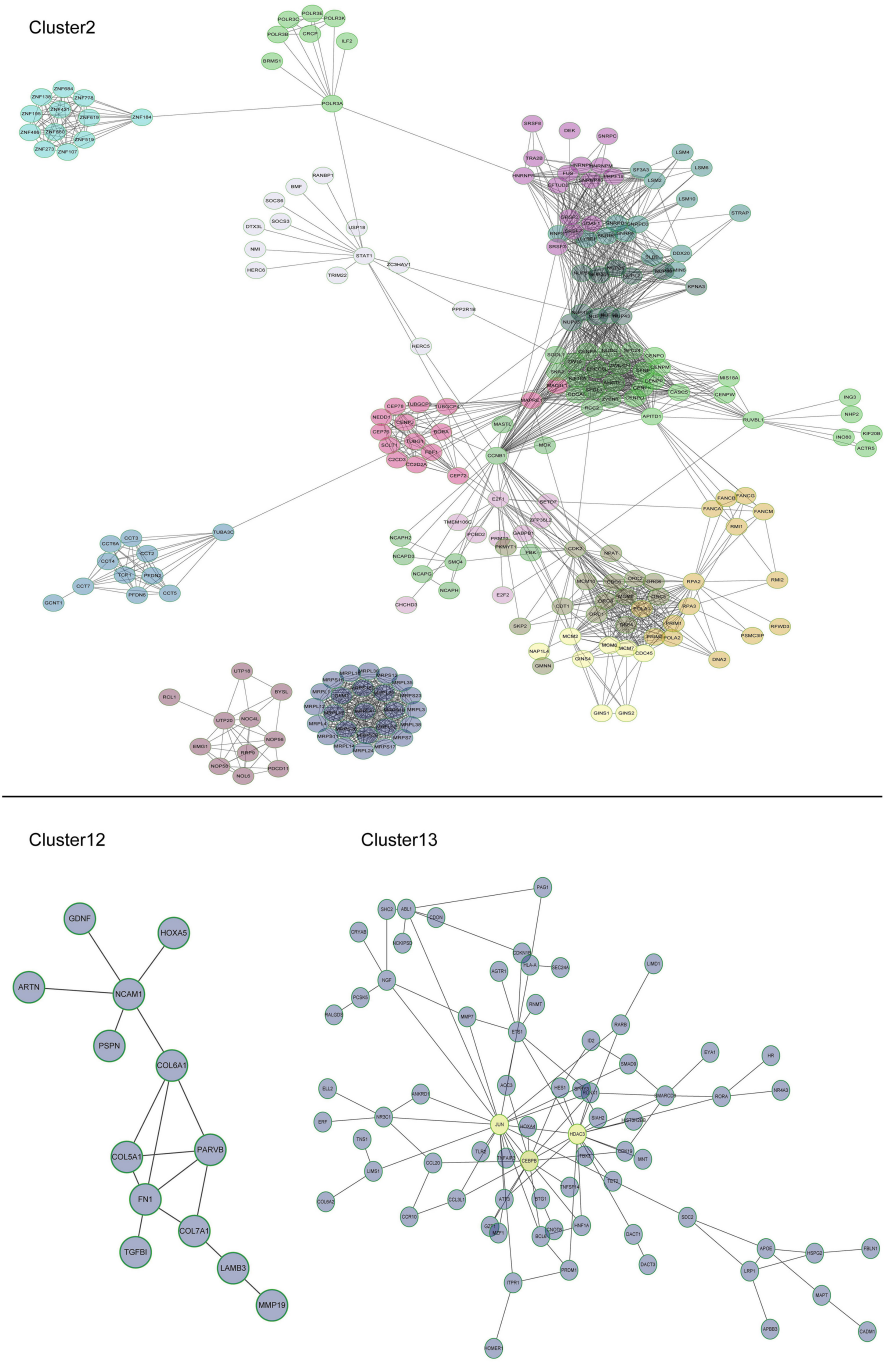
Subnetworks for U87-MG cell clusters. In cluster 2, 16 modules were produced, including mitochondrial ribosomal protein family, chaperonin containing TCP1, and cell cycle genes. A module was produced for clusters 12 and one for cluster 13. JUN, CEBPB, and HDAC3 were induced by 1% O_2_.

Multi-gene signatures are more effective than single gene expression values in order to investigate the relevant cellular phenotype. A network-module based method was performed for genes in cluster groups of U87-MG cells along with their expression values in order to identify hypoxia response specific gene interaction modules. Each module comprised a set of genes that were both topologically close to the functional interaction network, and correlated highly with regard to their expression levels. In cluster 2, 16 modules were produced, including mitochondrial ribosomal protein family, chaperonin containing TCP1 and cell cycle genes, whereas only one module was present for clusters 12 and 13. A total of 13 and 72 genes were filtered in clusters 12 and 13, respectively (Figure 5).

**Figure 6:**
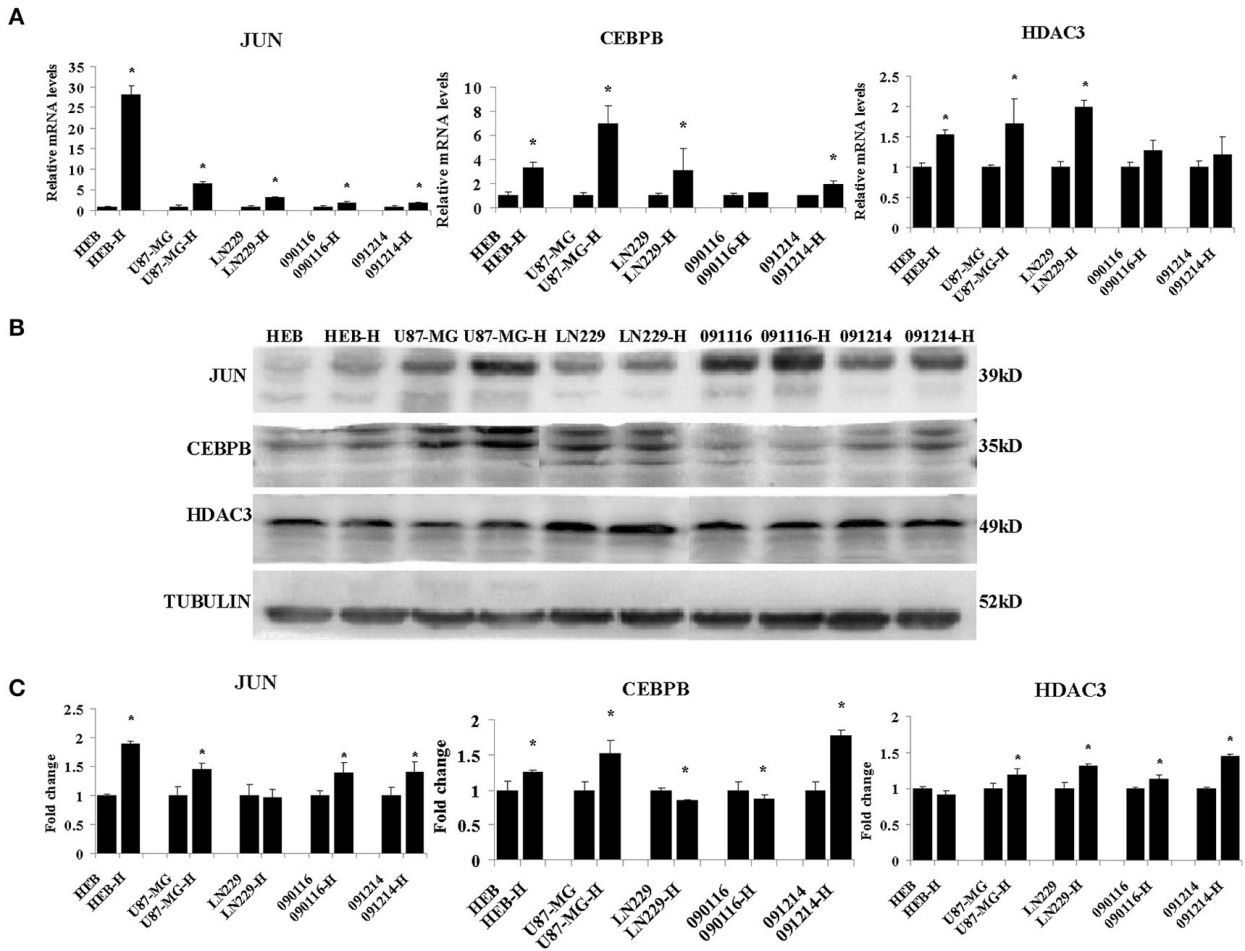
The mRNA **(A)** and protein **(B)** expression levels of JUN, CEBPB and HDAC3 in HEB, U87-MG, LN229, 091116, and 091214 cells following incubation of the cells under 21% O_2_ and 1% O_2_ conditions (H) for 24 (qPCR) or 48 h (western blot). **(C)** The quantitative analysis of protein bands in **(B)**, normalized to corresponding normoxic samples. All experiments were independently repeated three times. The data of experiments were presented as mean ± SD. *p < 0.05 vs. the control group.

**Figure 7:**
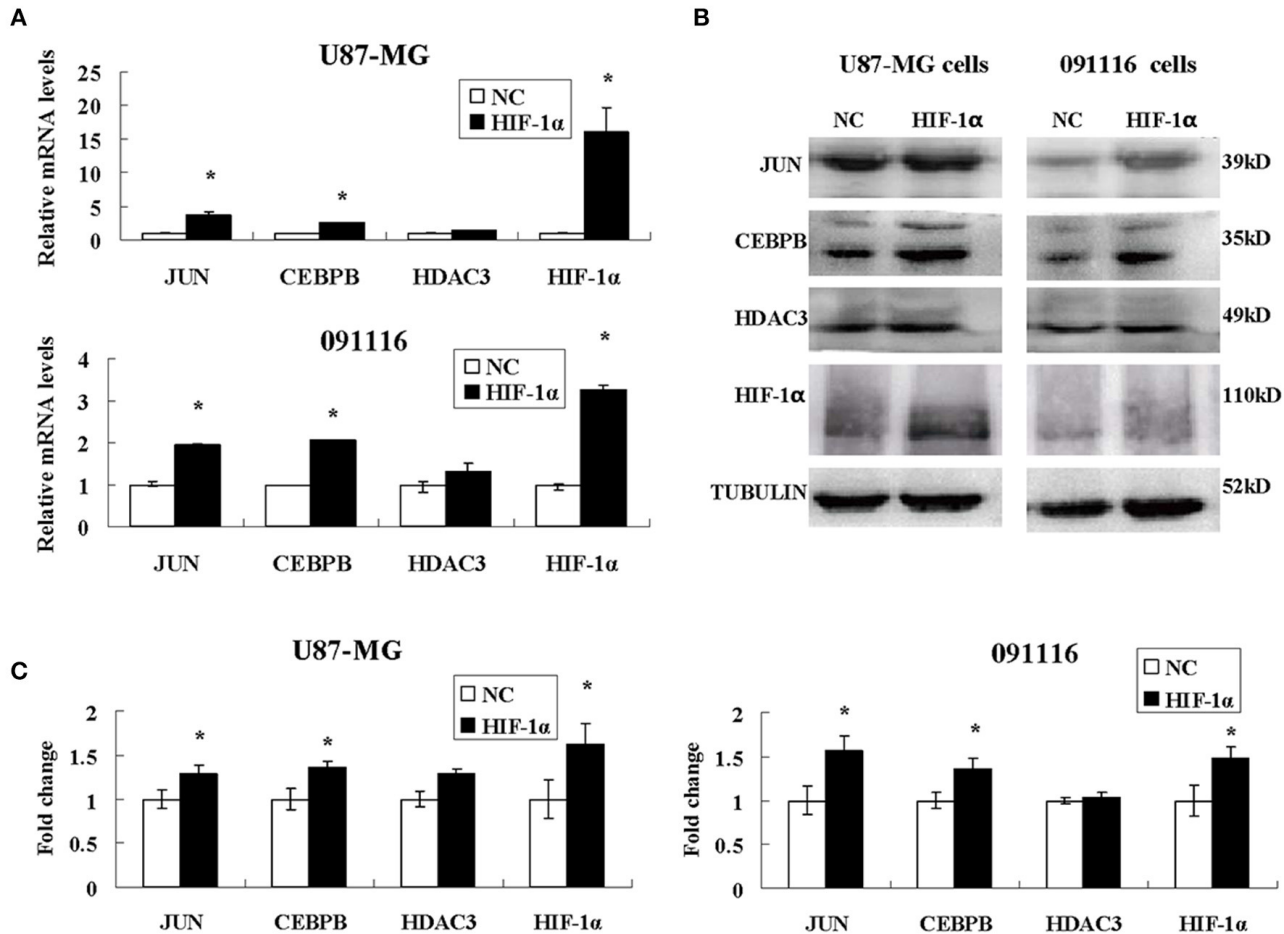
The expression levels of JUN, CEBPB and HDAC3 in U87-MG and 091116 cells following HIF-1α transfection. The mRNA levels of JUN and CEBPB mRNA in U87-MG and 091116 cells **(A)** were significantly increased. The levels of JUN and CEBPB protein in U87-MG and 091116 cells **(B)** were significantly increased. **(C)** The quantitative analysis of protein bands in **(B)**, normalized to negative control. All experiments were independently repeated three times. The data of experiments were presented as mean ± SD. *p < 0.05 vs. the negative control (NC) group.

Among the different genes involved in tissue and system development, jun proto-oncogene (JUN), transcriptional factors CCAAT/enhancer binding protein beta (CEBPB), and histone deacetylase 3 (HDAC3) were induced by extreme hypoxia (1% O_2_). The expression values of these genes correlated with the expression levels of additional genes in the cluster. Additionally, the results of qPCR (Figure 6A) and western blotting (Figures 6B,C) experiments further confirmed that hypoxia (1% O_2_) could promote the induction of JUN, CEBPB, and HDAC3 in GBM cells. Moreover, following overexpression of HIF-1α in U87-MG and 091116 cells, JUN, CEBPB, and HDAC3 were upregulated (Figure 7), which implied that these genes were downstream targets of HIF-1α. These observations indicated that JUN, CEBPB, and HDAC3 could play a central role in the regulation of other genes associated with GBM survival.

### JUN, CEBPB, and HDAC3 Involved in the Drug Resistance Phenotype of Hypoxic GBM

**Figure 8:**
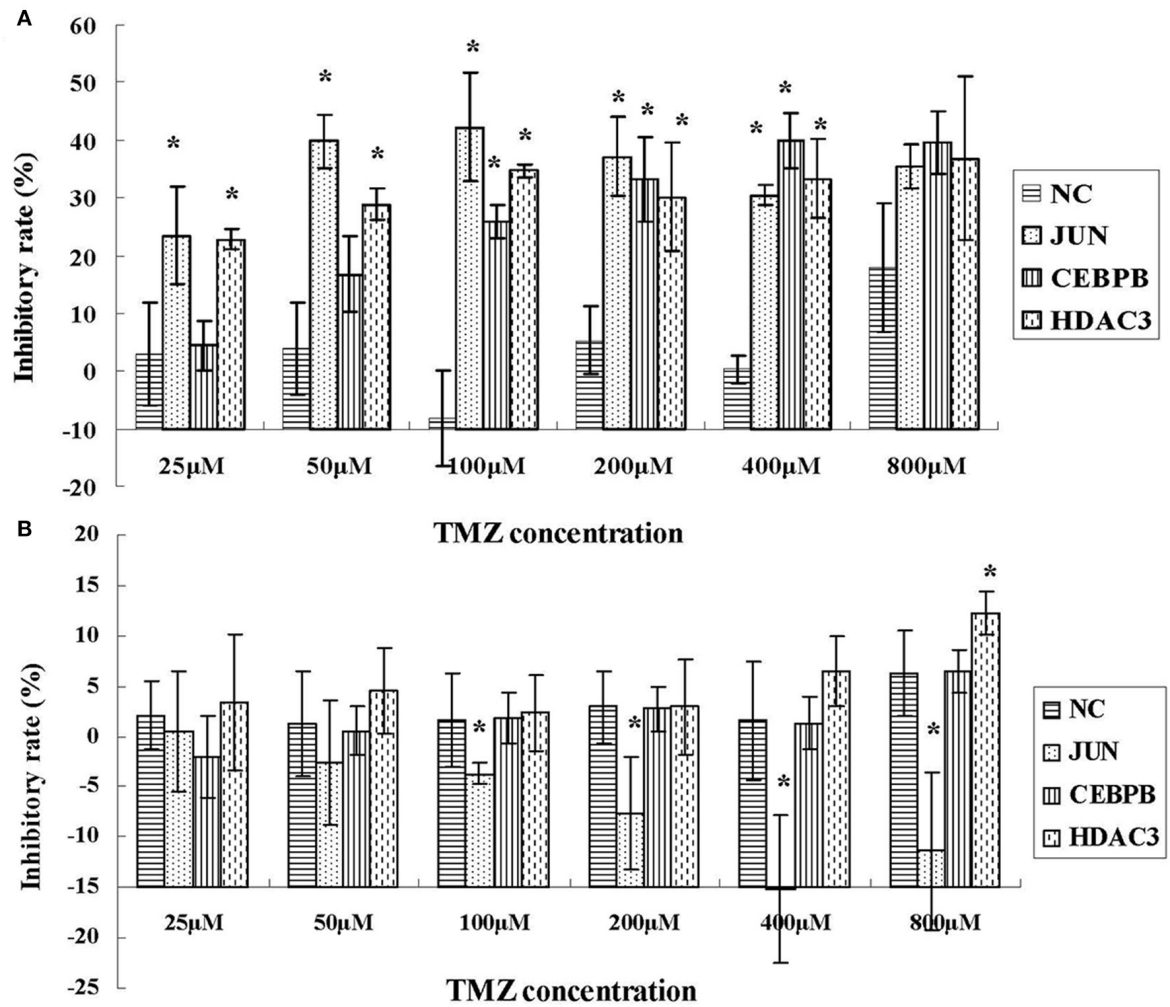
The inhibitory effects of TMZ in hypoxic U87-MG cells **(A)** and HEB cells **(B)** following knockdown of JUN, CEBPB, and HDAC3 by siRNA. All experiments were independently repeated three times. All data are presented as mean ± SD. *p < 0.05 vs. the negative control (NC) group (scramble siRNA).

To investigate the role of JUN, CEBPB and HDAC3 in the drug resistance phenotype of hypoxic GBM, we detected the inhibitory effects of temozolomide (TMZ) in four different GBM cell lines, namely U87-MG, LN229, 090116, and 091214. HEB cells were used as control. Temozolomide is a DNA methyltransferase inhibitor used for GBM treatment (20). However, the results suggested that TMZ could not induce cytotoxic effects in hypoxic U87-MG cells until a concentration of 800 μM was used (Figure 8A). However, JUN, CEBPB, and HDAC3 knockdown (Supplementary Table 1 and Supplementary Figure 1) resulted in significant inhibition of cell proliferation caused by low TMZ concentrations (25, 50, 100, and 200 μM), which was considerably lower than that noted at 800 μM (Figure 8A).

**Figure 9:**
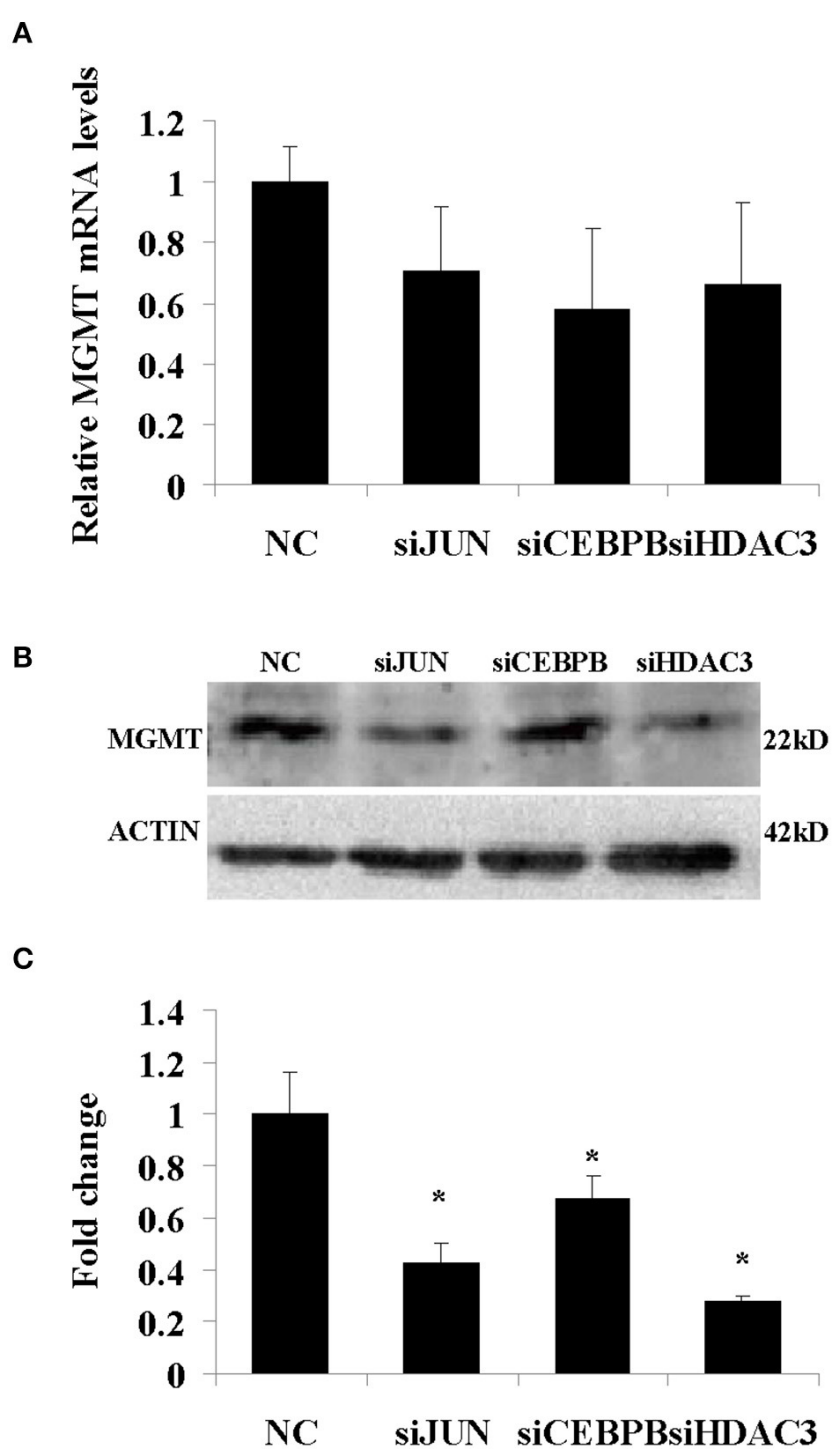
The mRNA **(A)** and protein **(B)** expression levels of MGMT following knockdown of JUN, CEBPB, and HDAC3 in U87-MG cells incubated under 1% O_2_ conditions. **(C)** The quantitative analysis of protein bands in **(B)**, normalized to negative control (NC). All experiments were independently repeated three times. All data are presented as mean ± SD. *p < 0.05 vs. the negative control (NC) group (scramble siRNA).

In addition, the results derived from LN229, 091116, and 091214 cells (Supplementary Figure 2) further validated the roles of JUN, CEBPB, and HDAC3 in regulating the drug resistance phenotype of hypoxic GBM cells. In contrast to the GBM cell lines, the proliferation of HEB cells did not show significant inhibition following downregulation of these genes (Figure 8B). Moreover, upon JUN, CEBPB and HDAC3 knockdown, the expression levels of MGMT decreased significantly (Figure 9), suggesting that these genes could regulate drug resistance of glioblastoma by mediating MGMT status. In conclusion, JUN, CEBPB and HDAC3 played a key role in the resistance to TMZ of hypoxic GBM cells by regulating MGMT.

## Discussion

Hypoxia plays a vital role in GBM progression and its therapeutic application has been validated by cell and animal models (11, 21). The comprehensive understanding of the altered complex cellular responses induced by hypoxia requires the detailed analysis of global molecular expression profiling. The present study explored the gene expression network of GBM cells induced by graded hypoxia by high-throughput RNA sequencing. We identified 3,341 genes that were clustered in 6 profiles and displayed significant changes in their expression levels following stimulation of hypoxia. The results further revealed that the genes with functions related to DNA replication and cell cycle were suppressed, while the genes involved in tissue and system development were activated following hypoxia. Although the changes were not complete in accordance with the biological function alteration, further studies are required that can examine the interpretation of these changes and identify the key pathways that are involved.

Recent reports have revealed that hypoxia is a critical regulator of the GBM microenvironment and that it is closely associated with resistance to various therapies (22). However, the mechanisms responsible for hypoxic survival of GBM cells remain unclear. In the present study, we demonstrated that hypoxia-induced repression of numerous genes was associated with DNA replication and cell cycle. Among these genes, the mitochondrial ribosomal protein family participated in energy production and mitochondrial disease (23), the chaperonin family containing TCP1 genes was implicated in cell cycle progression (24), and cyclin B1 (CCNB1) as a cell cycle regulated protein was involved in cell proliferation and tumor growth (25). The majority of anti-cancer drugs exert their function by inhibiting or damaging cell cycle events (26, 27). These drugs can exhibit decreased efficacy when the cell cycle process of GBM is suppressed under hypoxic conditions.

The data demonstrated that the expression levels of several genes associated with tissue and system development were increased under hypoxia, notably in the presence of 1% O_2_. JUN, CEBPB, and HDAC3 played a central role in the functional module. This demonstrated that extreme hypoxia could trigger the changes noted in the expression pattern of the selected GBM genes via the activation of transcription factors and epigenetic regulators. Transcription factors are the key effectors of eukaryotic gene control. JUN is a protooncogene that plays a critical role in cell proliferation and malignant transformation with its levels are elevated in GBM (28). It is interesting to note that previous work has reported the ability of JUN to promote drug resistance through MGMT (29) and MDR1 (30). CEBPB was identified as the epigenetic regulator of the mesenchymal signature and was used to predict poor clinical outcome (31). Coincidentally, CEBPB was involved in glioma progression by regulating cyclin D1 (32), and was highly expressed in glioblastoma stem cells, which also exhibited distinct resistance to chemotherapy (33). Histone deacetylases regulate the expression and activity of numerous proteins involved in both cancer initiation and progression (34, 35). HDAC3 is a class I histone deacetylase that acts as a critical regulator of gene expression via maintaining chromatin structure and genome stability (36). This enzyme presented higher expression levels in breast and gastric cancers and acute lymphoblastic leukemia cells and correlated with a poor patient prognosis (37–39). Recently, Li et al. reported that the HDAC3 inhibitor RGFP109 could overcome temozolomide resistance in glioblastoma cells. Moreover, HDAC3 could regulate MRP expression by MYCN (40, 41). All these studies suggested that JUN, CEBPB, and HDAC3 could promote the drug resistant phenotype of GBM by multiple pathways. Therefore, we investigated the proliferation of GBM in the presence or absence (knockdown) of these genes. The cytotoxic assay of TMZ implied that these three genes played a significant role in the drug resistance phenotype of hypoxic GBM cells. Furthermore, they affected the biological function of GBM via the regulation of the MGMT expression. In addition, JUN displayed a significant role in all four cell lines, which suggested that targeting JUN could be an effective strategy to overcome the GBM drug resistant phenotype.

**Figure 10:**
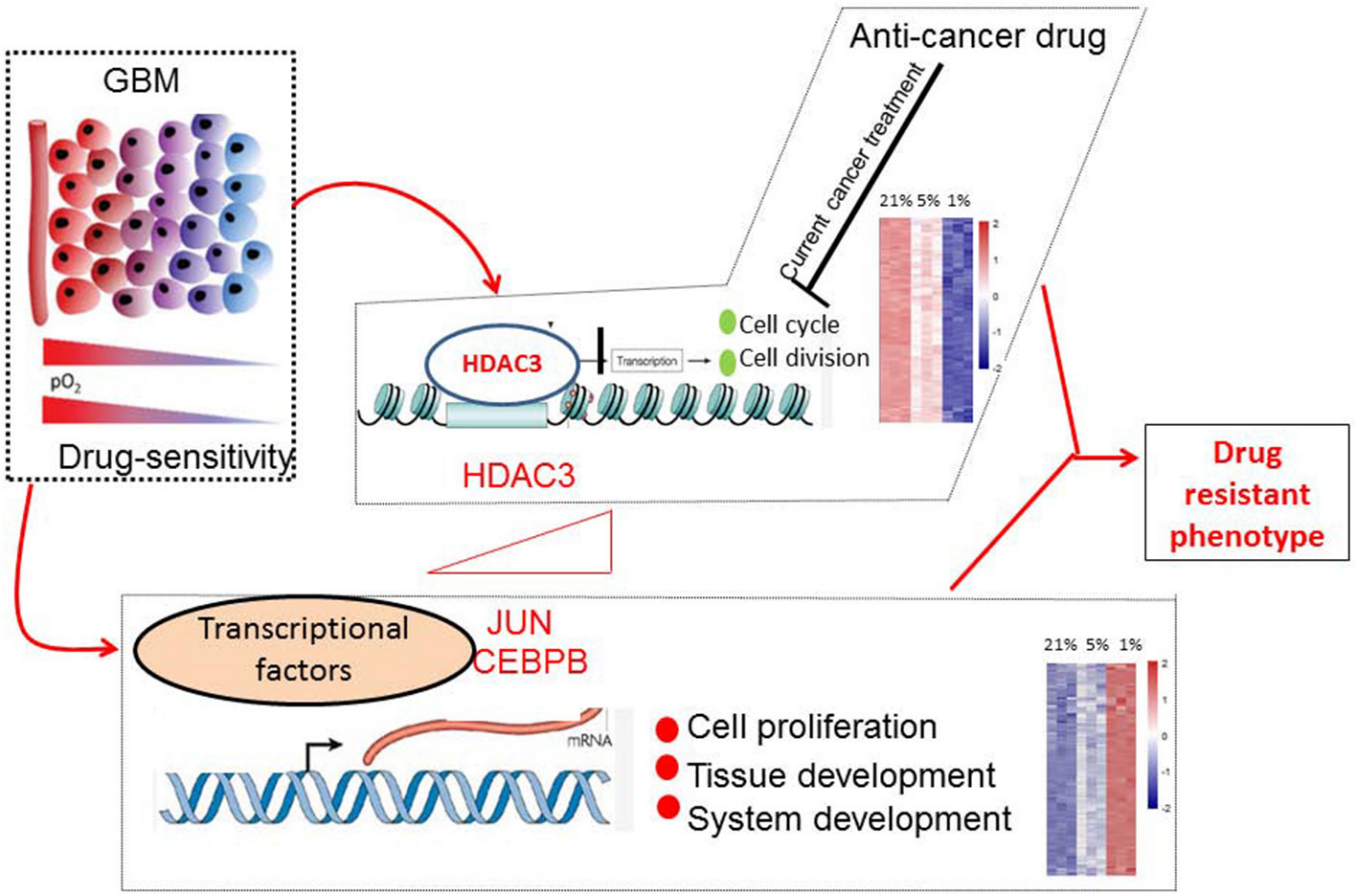
Molecular mechanisms of drug-resistant phenotype in hypoxic GBM cells. Hypoxia stimulated the expression of JUN, CEBPB, and HDAC3. The transcription factors JUN and CEBPB activated the genes involved in tissue and system development. HDAC3 selectively silenced the genes associated with cell cycle progression.

In conclusion, we monitored with unprecedented resolution the hypoxic GBM cell transcriptome. Our work has demonstrated that the molecular mechanisms of the drug-resistance phenotype of hypoxic GBM can be summarized in two major domains. One included the activated transcription factors JUN and CEBPB, which were notably involved in tumorigenesis. The second domain involved the genes responsible for the suppression of cell cycle events that were targeted by HDAC3 (Figure 10). Exploring the detail targets of JUN, CEBPB, and HDAC3 will reveal the drug resistance phenotype of hypoxic GBM and promote the potential therapeutic applications for this disease.

## Materials and Methods

### Cell Culture and Hypoxic Treatment

The human normal glial cell line, HEB, was generously provided by Professor Guang-mei Yan (Department of Pharmacology, Sun Yat-sen University, Guangzhou, China) (42). The human GBM cell lines U87-MG and LN229 were obtained from the American Type Culture Collection. The 090116 and 091214 cell lines were primary GBM cell lines derived from the Institute of Pathology and Southwest Cancer Center, at the Southwest Hospital, in the Army Medical University of Chongqing in China. These cells were incubated in DMEM supplemented with 10% fetal bovine serum (FBS), 2 mM L-glutamine, 100 U/ml penicillin, and 100 μg/ml streptomycin for 24 h at normoxia (21% O_2_) and subsequently for another 24 h under normoxic or hypoxic conditions (5 or 1% O_2_, respectively) in hypoxic chambers (Thermo scientific), respectively.

### Cell Growth Assay

The cell proliferation assay was carried out with the Cell Couting Kit-8 (CCK-8, Dojindo, Kumamoto, Japan) according to the manufacturer's instructions. U87-MG and HEB cells were planted in 96-well plates (5,000 cells per well with 100 μl growth medium) and cultured in a hypoxic chamber followed by 24 h incubation a normoxic incubator. The cell viability was determined at 450 nm by a 96-well plate spectrophotometer (Multiskan GO, Thermo Scientific, USA).

### Cell Apoptosis Assay

The cell apoptosis was measured by the Annexin V-fluorescein isothiocyanate apoptosis detection kit (Keygen Biotech, Nanjng, China). Hypoxic U87-MG and HEB cells were collected, centrifuged, washed with phosphate-buffered saline and counted with an electronic cytometer (Beckman Coulter, Miami, FL). Approximately 1.0 × 10^5^ cells were resuspended in 190 μl of Annexin V-fluorescein isothiocyanate-binding buffer, and subsequently 5 μl of Annexin V-fluorescein isothiocyanate and 5 μl of propidium iodide were added. The samples were incubated for an additional 10 min with the samples in dark at room temperature. The fluorescence of the cells was detected and the results were analyzed by flow cytometry.

### Cell Cycle Assay

The analysis of the cell cycle was performed by an Accuri 6 flow cytometer (Accuri Cytometers, Inc., Ann Arbor, MI, USA) and by the Cell Quest software following the manufacturer's protocols. Hypoxic U87-MG and HEB cells were collected, centrifuged and fixed with 70% ethanol. The samples were analyzed by flow cytometry.

### mRNA-Seq Library Preparation and Sequencing

Total RNA was extracted with the Trizol reagent and quantified using a Nanodrop ND-1000 spectrophotometer. RNA integrity was verified on an Agilent 2100 Bioanalyzer and the RIN was listed in Supplementary Table 2. Illumina mRNA-seq libraries were prepared using the TruSeq RNA kit by 200 ng of total RNA. The library was sequenced on an Illumina HiSeq™ 2000 sequencing apparatus. RNA-seq reads were mapped using Bowtie2 (version) (43) against the human genome build hg19 with specific settings of the corresponding parameters (-q –phred64 –sensitive –dpad 0 –gbar 99999999 –mp 1,1 –np 1 –score-min L,0,-0.1 -I 1 -X 1000 –no-mixed –no-discordant -p 16 -k 200). The FPKM (Fragments Per Kilobase Of Exon Per Million Fragments Mapped) method was used to estimate the expression levels of genes, calculated by Expectation Maximization (RSEM) (44). A principal component analysis (PCA) was performed using function fast.prcomp in gmodels package based on the R platform in order to determine the interrelations between the individual samples. The analysis was conducted using the normalized counts of all the genes after filtering the low expression level genes as the input. The sequential expression profiles derived from STEM software were applied to detect the statistically enriched gene families within each profile. The software assumes that the experiments can naturally be sequentially ordered (45). The overall genes were assigned to the profiles and the *p*-values for each profile were computed based on a binominal distribution and subsequently corrected for multiple hypothesis testing using a FDR of <5%. The genes in each significantly expressed cluster were subjected to GO classifications using the gene ontology dataset (46). Cytoscape (47) was used to assess the biological significance for each of the obtained clusters by gene-based pathway and ontology analysis. Notably, the reactome functional interaction plugin was used to assess the membership of genes to the reactome and KEGG pathways were applied to calculate enrichment with a *p-*value corrected by the Bonferroni method (48).

### cDNA Synthesis and qPCR

RNA from cells was isolated using the total RNA Kit I (Takara, R6834-02) according to the manufacturer's protocol. cDNA was synthesized using PrimeScript RT reagent kit (Takara, RR047A) with random primers for RT priming. qPCR was performed using SYBR Green (Bio-Rad, RR820A) according to the manufacturer's instructions.

### Protein Extraction and Western Blot

Whole cell lysates were obtained by re-suspending cell pellets in RIPA buffer (Beyotime, P0013E) with a freshly added protease inhibitor tablet (Thermo Scientific, 88265). Western blot analyses were performed with anti-β-actin (Kangcheng, KC-5A08), anti-α-Tubulin (Proteintech, 11224-1-AP), anti-JUN (Proteintech, 24909-1-AP), anti-CEBPB (Proteintech, 23431-1-AP), anti-HDAC3 (Proteintech, 10255-1-AP), anti-HIF-1α(Proteintech, 20960-1-AP), and anti-MGMT (Proteintech, 17195-1-AP) antibodies.

### Transient Transfection of HIF-1α Plasmids

U87-MG and 091116 cells were grown to 80–90% confluence in 6-well plates and transiently transfected using Lipofectamine 3000 (Invitrogen) with pcDNA3.1-HIF-1α (as well as the empty vector as negative control). The cells were collected for qPCR and western blot assays at 24 and 48 h, respectively.

### Temozolomide Treatment Assay

To analyze the role of JUN, CEBPB and HDAC3 in the drug resistance of hypoxic GBM, we transferred HEB, U87-MG, LN229, 091116, and 091214 cells in 96-well plates. Following 24 h of incubation, we transfected the cells with siRNA that resulted in knockdown of JUN, CEBPB, and HDAC3. The cells were further incubated in a 1% hypoxic chamber. Temozolomide (Meilunbio Company, Dalian, China) was added into the culture media 24 h later and the cell viability was detected following an additional 48 h culture in a 1% hypoxic chamber.

### Detection of MGMT Expression Levels

We transferred U87-MG cells in 6-well plates. Following 24 h incubation, we transfected the cells with siRNA that resulted in knockdown of JUN (homo-1949), CEBPB (homo-1703), and HDAC3 (homo-311). The resulting cells were incubated the cells in a 1% hypoxic chamber. The cells were collected for MGMG mRNA and protein detection following 48 h of hypoxic treatment.

### Statistical Analysis

Statistical analysis was carried out using the SPSS 19.0 software. CCK-8, apoptosis and cell cycle assays were analyzed using the one-way ANOVA test. The remaining experiments were analyzed using the independent samples *t*-test. A value of *p* < 0.05 was considered to indicate a significant result. Statistical LSD tests were performed among 21, 5, and 1% O_2_ conditions. All experiments were independently carried out in triplicate.

### Data Accession

The raw data have been deposited to the Gene Expression Omnibus (GEO) under accession GSE78025.

## Author Contributions

YiG designed the study, performed experiments, interpreted the data, and wrote the manuscript. BL and CL were involved in the biological informatics analysis. LF, SH, BS, and GE participated in the cellular experiments. YY completed the western blot experiments, analyzed the data and revised the manuscript. EZ and BZ conceived the study design, experimental plan, and manuscript writing. YuG participated in manuscript writing. GW participated in the figure drawing. All authors discussed the results and critically reviewed the manuscript.

### Conflict of Interest Statement

The authors declare that the research was conducted in the absence of any commercial or financial relationships that could be construed as a potential conflict of interest.

## Supplementary Material

Supplementary Figure 1 The results of qPCR and western blot for siRNA transfection, **(A)** and **(B)** for JUN, **(C)** and **(D)** for CEBPB, **(E)** and **(F)** for HDAC3. All experiment was repeated three times independently. All data are presented as mean ± SD.

Supplementary Figure 2 The inhibitory effect of TMZ in hypoxic LN229 **(A)**, 091116 **(B)** and 091214 cells with JUN, CEBPB and HDAC3 knocked down by siRNA. All experiments were independently repeated three times. All data are presented as mean ± SD. ^*^, *p* < 0.05 versus the NC group.

Supplementary Figure 3 The splicing data and the raw images of Figure 6B. Red arrows represent the locations of protein markers and green arrows represent the locations of splicing.

Supplementary Figure 4 Repeated experiments of Figure 6B without splicing. The protein expression levels of JUN, CEBPB and HDAC3 in HEB, U87-MG, LN229, 091116, and 091214 cells following incubation of the cells under 21% O_2_ and 1% O_2_ conditions (H) for 48 h.

Supplementary Table 1 Sequences of siRNA for knockdown of JUN, CEBPB and HDAC3.

Supplementary Table 2 The RIN for samples.
