# Corrigendum: Targeting JUN, CEBPB, and HDAC3: A Novel Strategy to Overcome Drug Resistance in Hypoxic Glioblastoma

**DOI:** 10.3389/fonc.2020.00644

**Published:** 2020-04-28

**Authors:** Yixing Gao, Bao Liu, Lan Feng, Binda Sun, Shu He, Yidong Yang, Gang Wu, Guoji E, Chang Liu, Yuqi Gao, Erlong Zhang, Bo Zhu

**Affiliations:** ^1^Department of Oncology, Xinqiao Hospital, Army Medical University, Chongqing, China; ^2^Institute of Medicine and Equipment for High Altitude Region, College of High Altitude Military Medicine, Army Medical University, Chongqing, China; ^3^Key Laboratory of High Altitude Medicine, People's Liberation Army, Chongqing, China

**Keywords:** glioblastoma, hypoxia, drug resistance, JUN, CEBPB, HDAC3

In the original article, there was an error in the accession number where the data was deposited. Instead of “GSE75665” it should be “GSE78025.” A correction has been made to the Materials and Methods section, subsection Data Accession:

“The raw data have been deposited to the Gene Expression Omnibus (GEO) under accession GSE78025.”

The apologize for this error and declared that this correction does not change the scientific conclusions of the article in any way. The original article has been updated.

