# Corrigendum: Targeting JUN, CEBPB and HDAC3: a novel strategy to overcome drug resistance in hypoxic glioblastoma

**DOI:** 10.3389/fonc.2024.1392899

**Published:** 2024-04-23

**Authors:** Yixing Gao, Bao Liu, Lan Feng, Binda Sun, Shu He, Yidong Yang, Gang Wu, Guoji E, Chang Liu, Yuqi Gao, Erlong Zhang, Bo Zhu

**Affiliations:** ^1^Department of Oncology, Xinqiao Hospital, Army Medical University, Chongqing, China; ^2^Institute of Medicine and Equipment for High Altitude Region, College of High Altitude Military Medicine, Army Medical University, Chongqing, China; ^3^Key Laboratory of High Altitude Medicine, People’s Liberation Army, Chongqing, China

**Keywords:** glioblastoma, hypoxia, drug resistance, JUN, CEBPB, HDAC3

In the published article, two Supplementary Figures were missing. We provide both the splicing data and the raw images of Figure 6B in Supplementary Figure 3. The Supplementary Figure 3 and caption have been added to the original article. Moreover, we have repeated the experiments of Figure 6B and provided the figure without splicing in Supplementary Figure 4. The Supplementary Figure 4 and caption have been added to the original article.

The authors apologize for these errors and state that this does not change the scientific conclusions of the article in any way. The original article has been updated.

