# Supplementary figures and images for: Targeting JUN, CEBPB, and HDAC3: A Novel Strategy to Overcome Drug Resistance in Hypoxic Glioblastoma
Source: Front Oncol. 2019 Feb 1;9:33. doi: 10.3389/fonc.2019.00033 (PMC6367651; doi:10.3389/fonc.2019.00033)

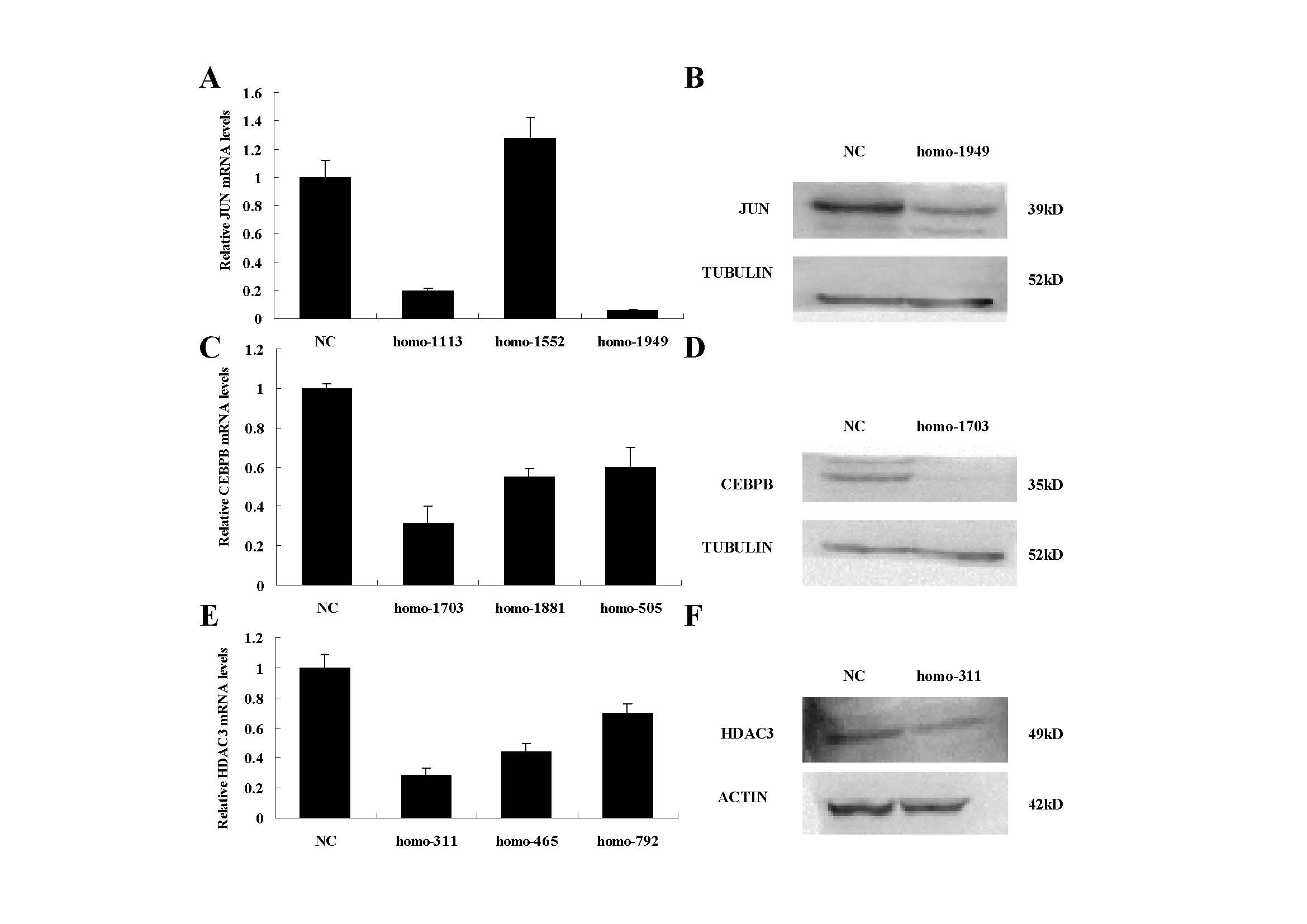

Supplement: Supplementary Figure 1 — The results of qPCR and western blot for siRNA transfection, (A) and (B) for JUN, (C) and (D) for CEBPB, (E) and (F) for HDAC3. All experiment was repeated three times independently. All data are presented as mean ± SD. [file Image_1.JPEG]

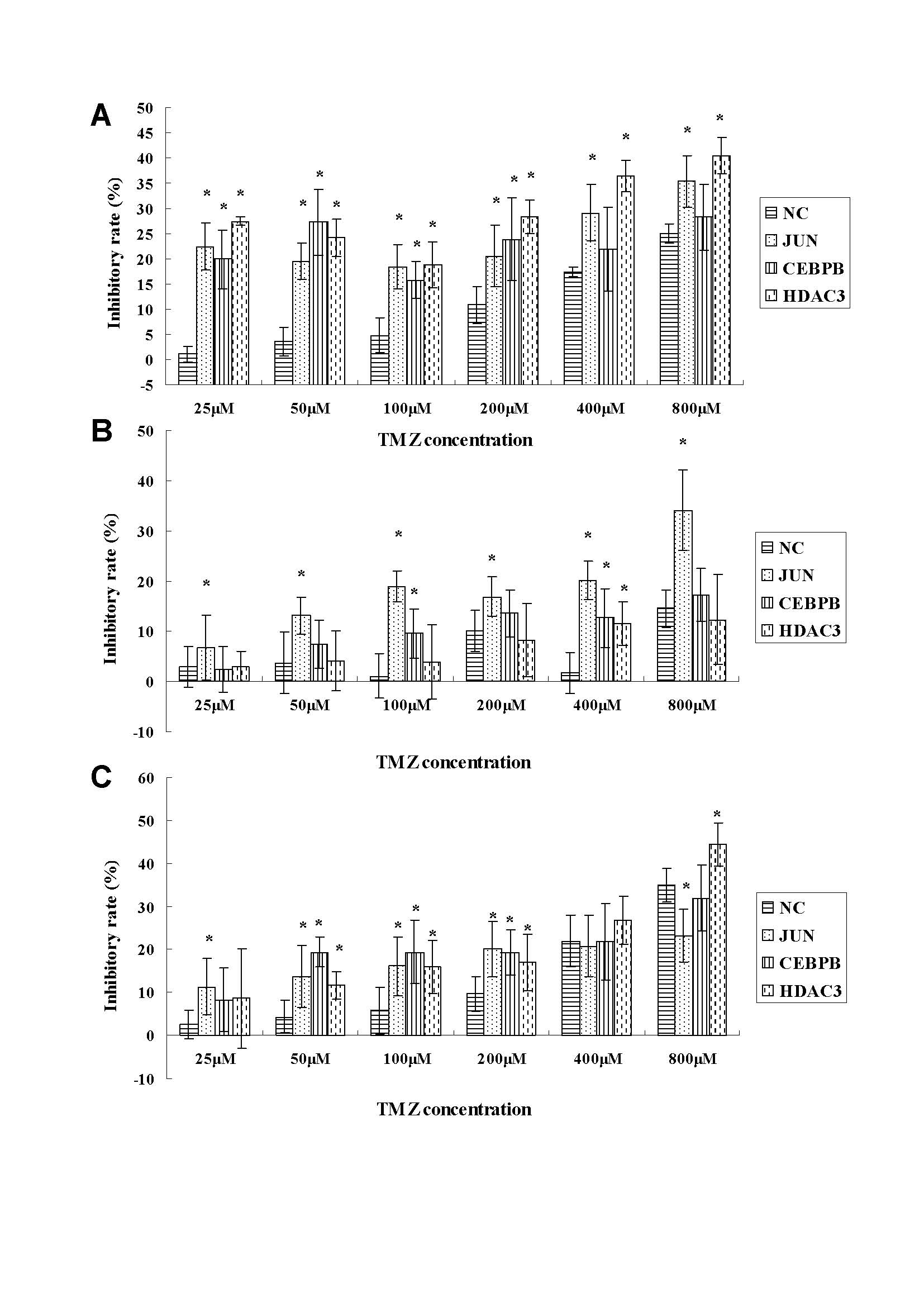

Supplement: Supplementary Figure 2 — The inhibitory effect of TMZ in hypoxic LN229 (A), 091116 (B) and 091214 cells with JUN, CEBPB and HDAC3 knocked down by siRNA. All experiments were independently repeated three times. All data are presented as mean ± SD. *, p < 0.05 versus the NC group. [file Image_2.JPEG]
