# Supplementary material for: Targeting JUN, CEBPB, and HDAC3: A Novel Strategy to Overcome Drug Resistance in Hypoxic Glioblastoma
Source: Front Oncol. 2019 Feb 1;9:33. doi: 10.3389/fonc.2019.00033 (PMC6367651; doi:10.3389/fonc.2019.00033)

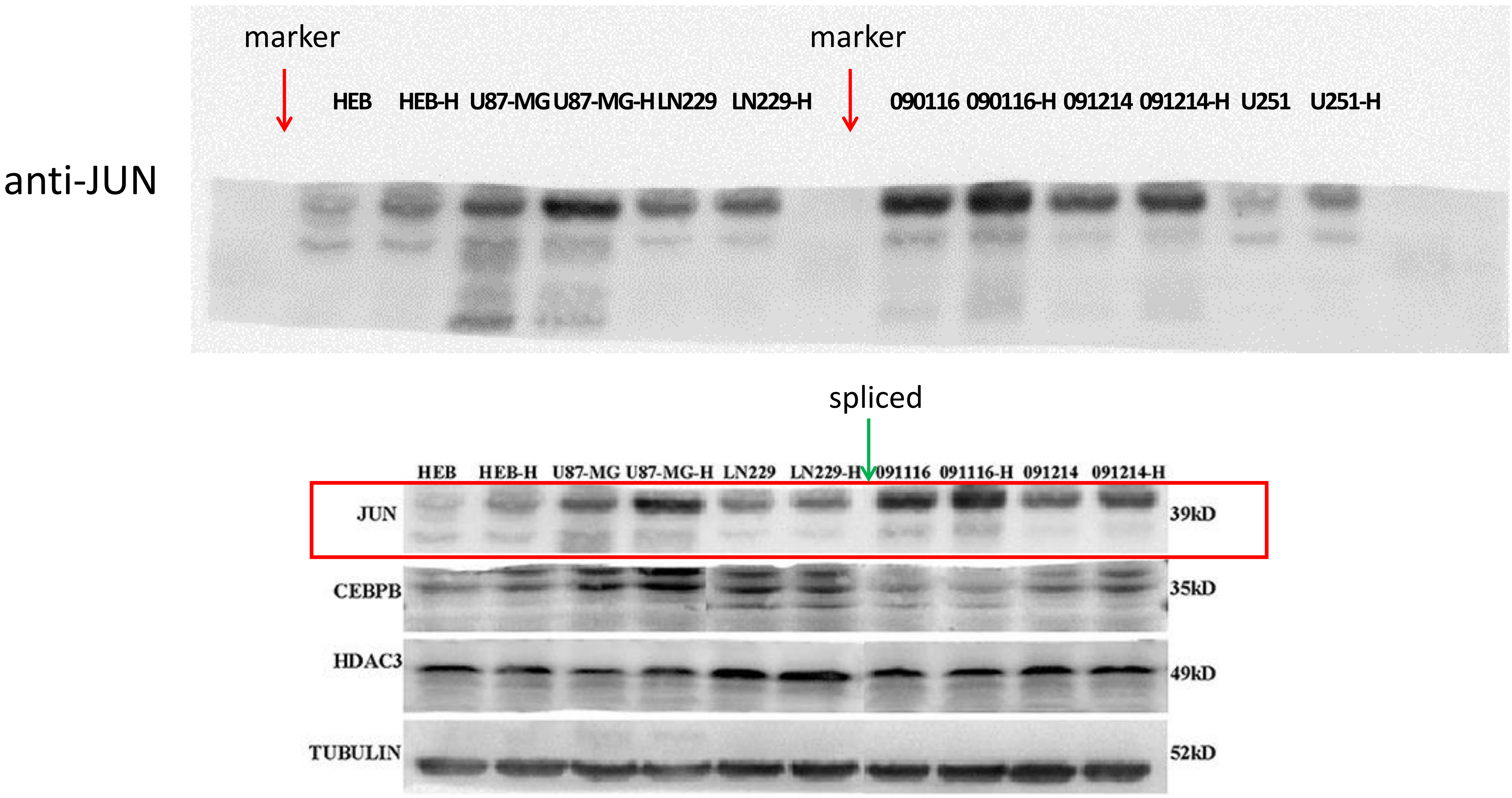

marker
marker
HEB HEB-H U87-MG U87-MG-H LN229 LN229-H
090116 090116-H 091214 091214-H U251 U251-H
anti-JUN
spliced
HEB HEB-H U87-MG U87-MG-H LN229 LN229-H 091116 091116-H 091214 091214-H
JUN
39kD
CEBPB
35kD
HDAC3
49kD
TUBULIN
52kD

anti-CEBPB

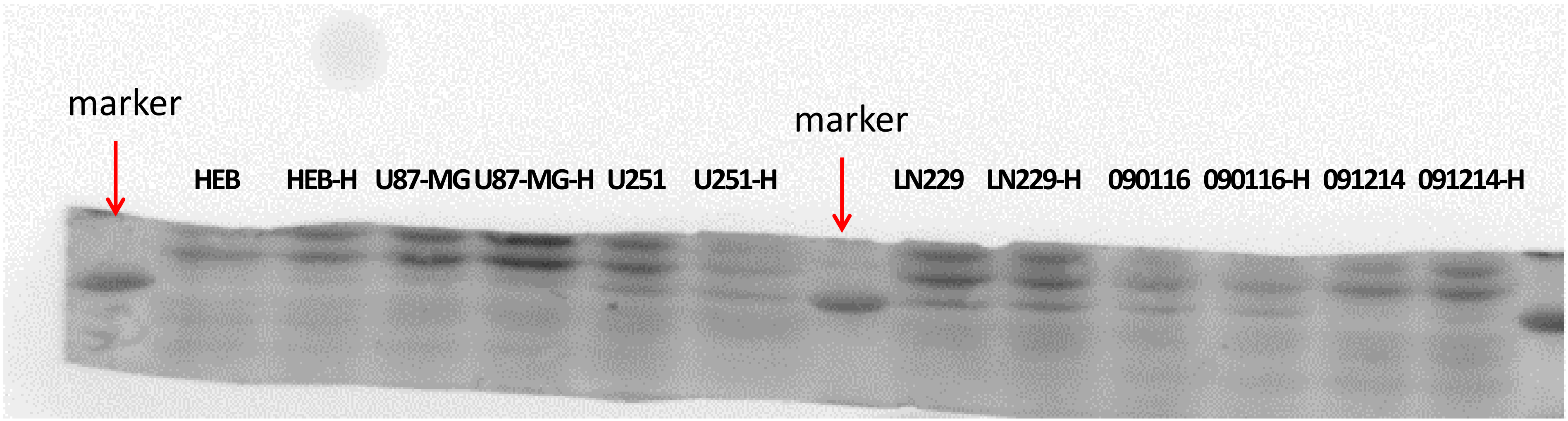
marker
marker
HEB
HEB-H
U87-MG
U87-MG-H
U251
U251-H
LN229
LN229-H
090116
090116-H
091214
091214-H

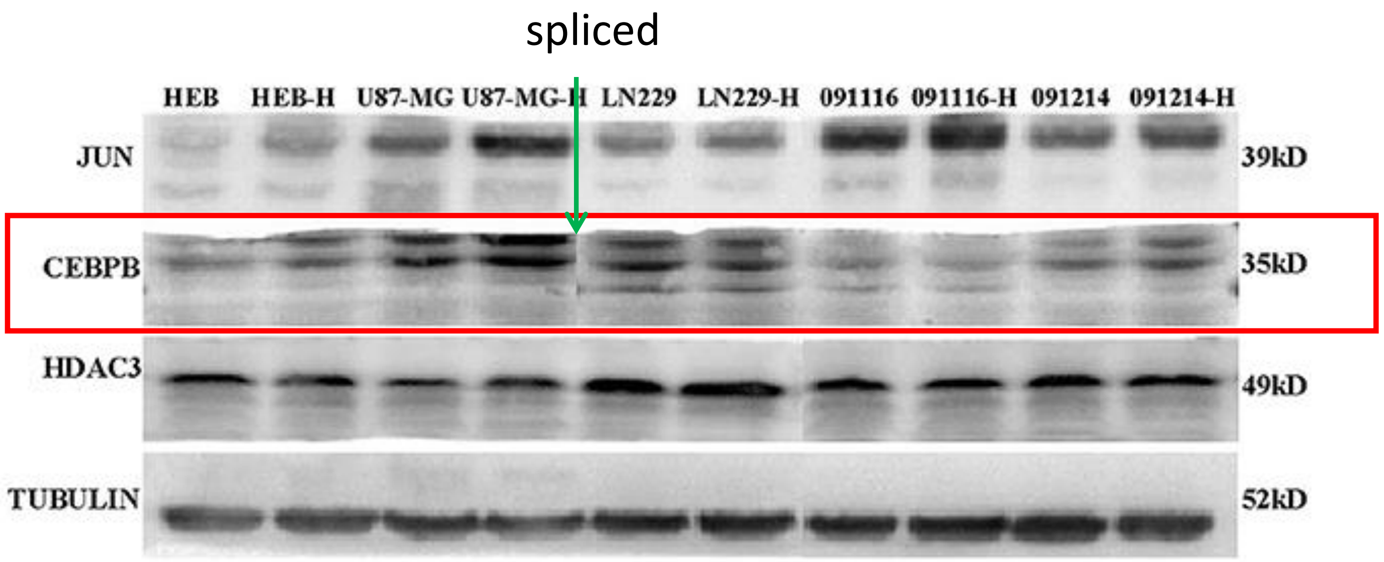
spliced
HEB
HEB-H
U87-MG
U87-MG-H
LN229
LN229-H
091116
091116-H
091214
091214-H
JUN
39kD
CEBPB
35kD
HDAC3
49kD
TUBULIN
52kD

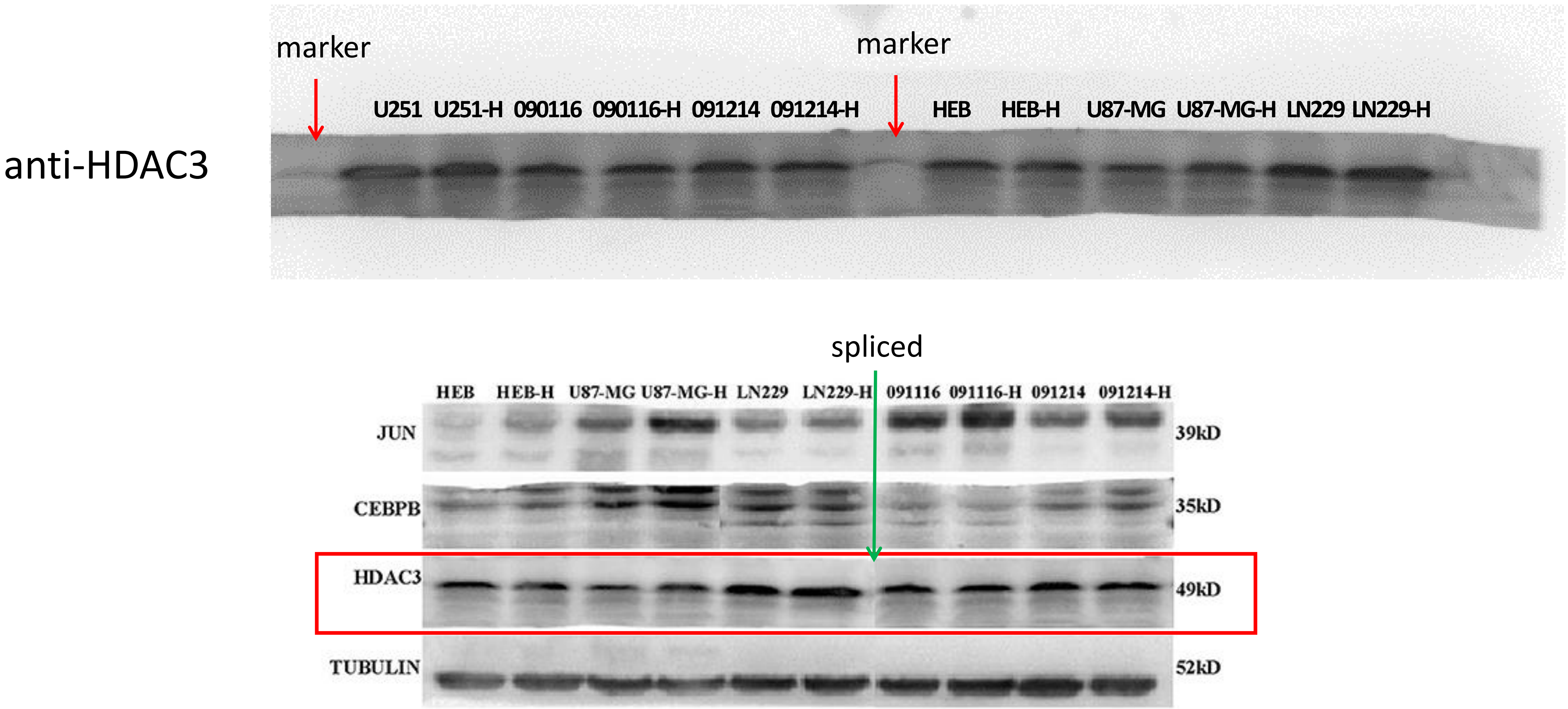
anti-HDAC3
marker
marker
U251 U251-H 090116 090116-H 091214 091214-H
HEB HEB-H U87-MG U87-MG-H LN229 LN229-H
spliced
HEB HEB-H U87-MG U87-MG-H LN229 LN229-H 091116 091116-H 091214 091214-H
JUN
39kD
CEBPB
35kD
HDAC3
49kD
TUBULIN
52kD

anti-TUBULIN

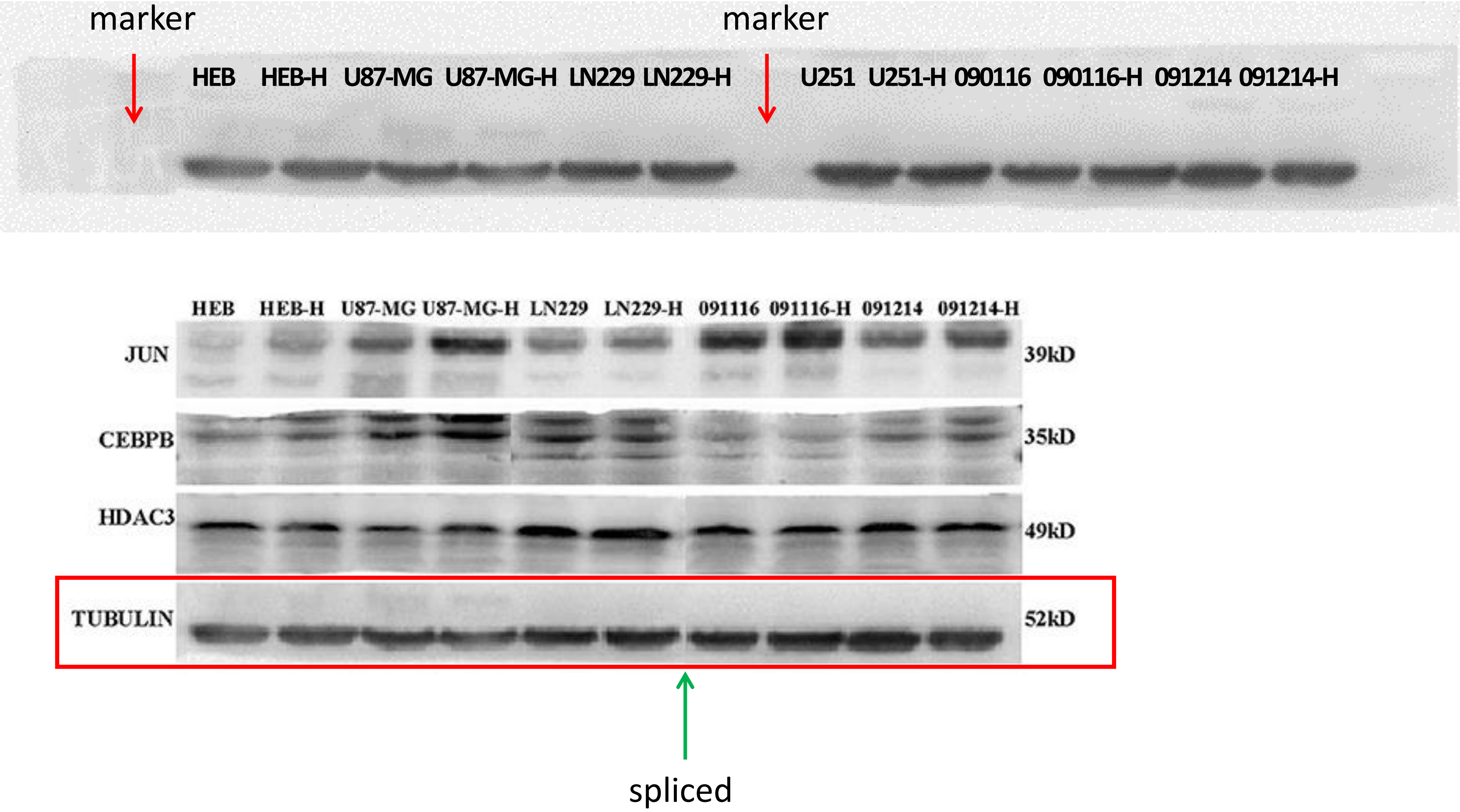

anti-JUN

HEB HEB-H U87-MG U87-MG-H U251 U251-H LN229 LN229-H 090116 090116-H 091214 091214-H

1st

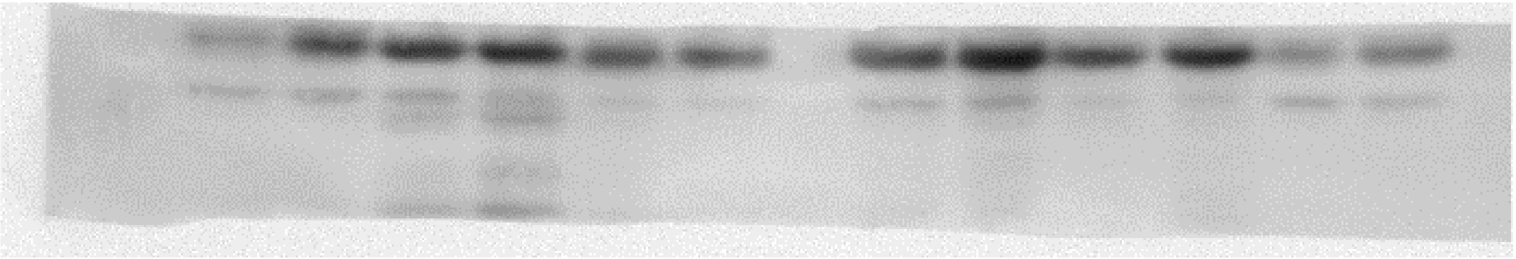

HEB HEB-H U87-MG U87-MG-H LN229 LN229-H 090116 090116-H 091214 091214-H U251 U251-H

2nd

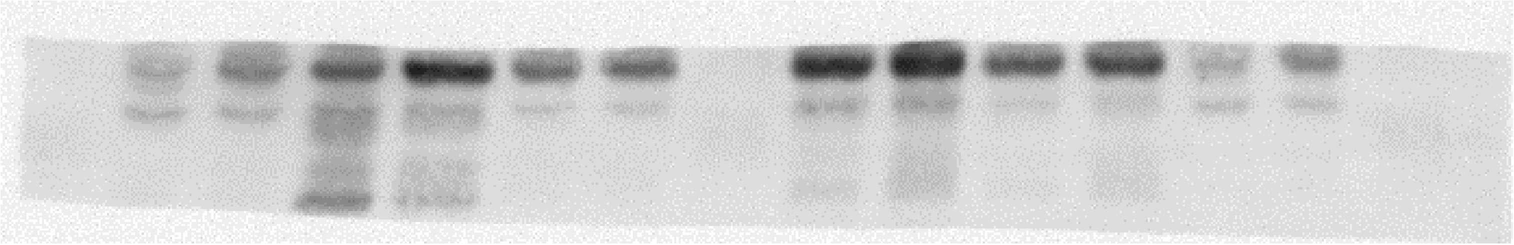

image in figure

U251 U251-H 090116 090116-H 091214 091214-H HEB HEB-H U87-MG U87-MG-H LN229 LN229-H

3rd

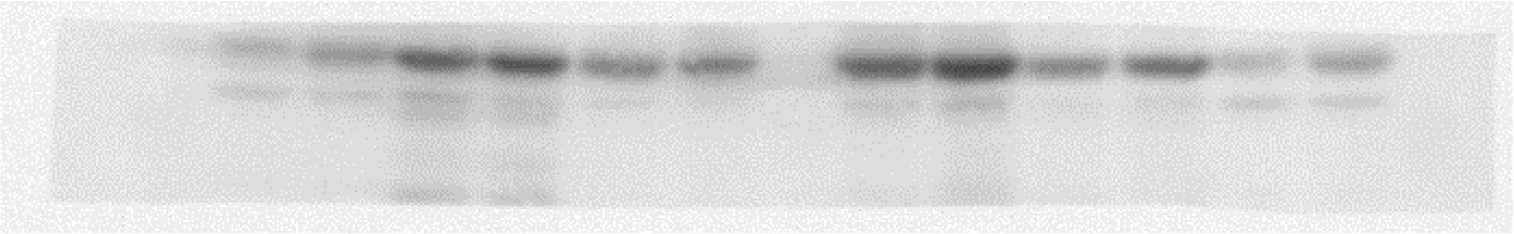

anti-CEBPB

1st

HEB HEB-H U87-MG U87-MG-H U251 U251-H LN229 LN229-H 090116 090116-H 091214 091214-H

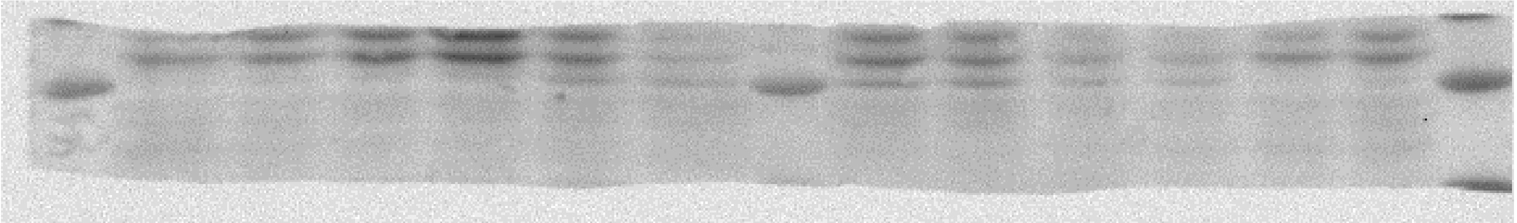

image in figure

2nd

HEB HEB-H U87-MG U87-MG-H LN229 LN229-H 090116 090116-H 091214 091214-H U251 U251-H

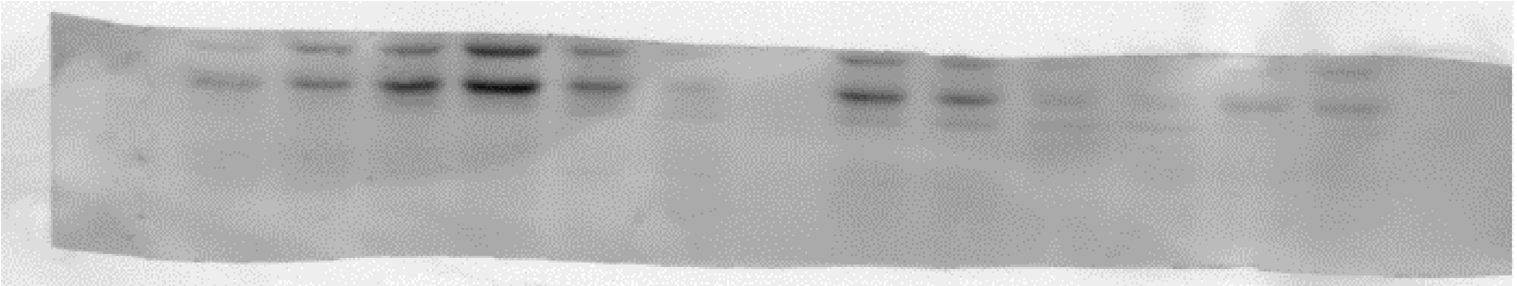

3rd

U251 U251-H 090116 090116-H 091214 091214-H HEB HEB-H U87-MG U87-MG-H LN229 LN229-H

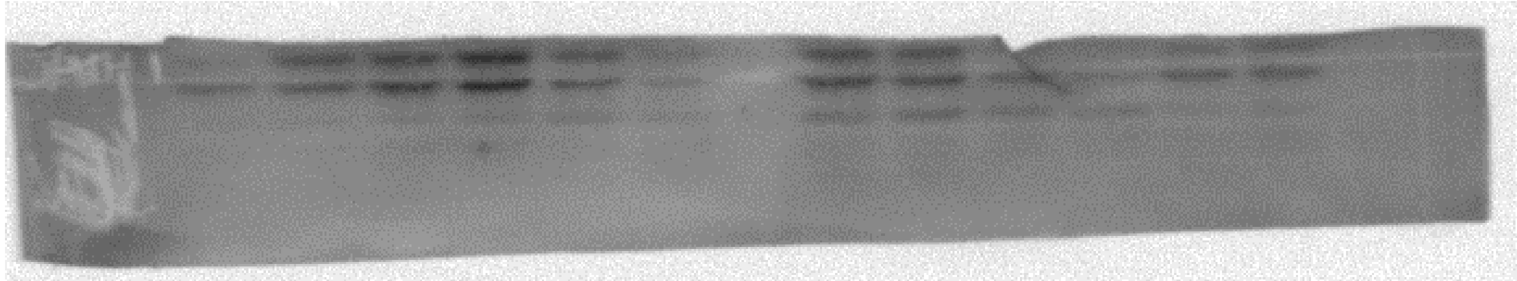

anti-HDAC3

HEB HEB-H U87-MG U87-MG-H U251 U251-H LN229 LN229-H 090116 090116-H 091214 091214-H

1st

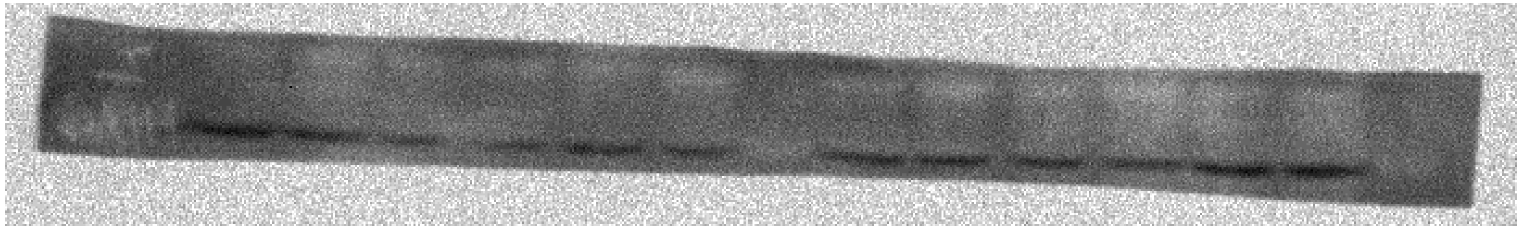

HEB HEB-H U87-MG U87-MG-H LN229 LN229-H 090116 090116-H 091214 091214-H U251 U251-H

2nd

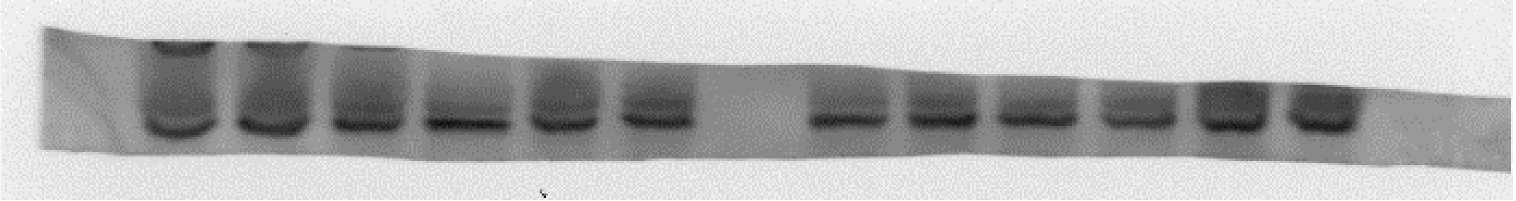

U251 U251-H 090116 090116-H 091214 091214-H HEB HEB-H U87-MG U87-MG-H LN229 LN229-H

3rd

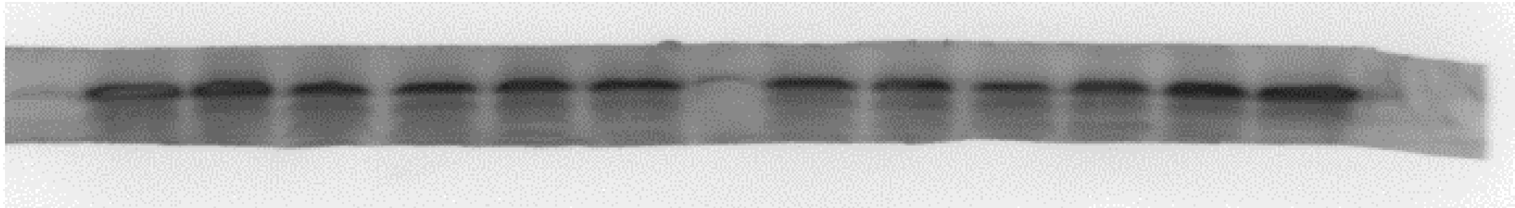

image in figure

anti-TUBULIN

HEB HEB-H U87-MG U87-MG-H U251 U251-H LN229 LN229-H 090116 090116-H 091214 091214-H

1st

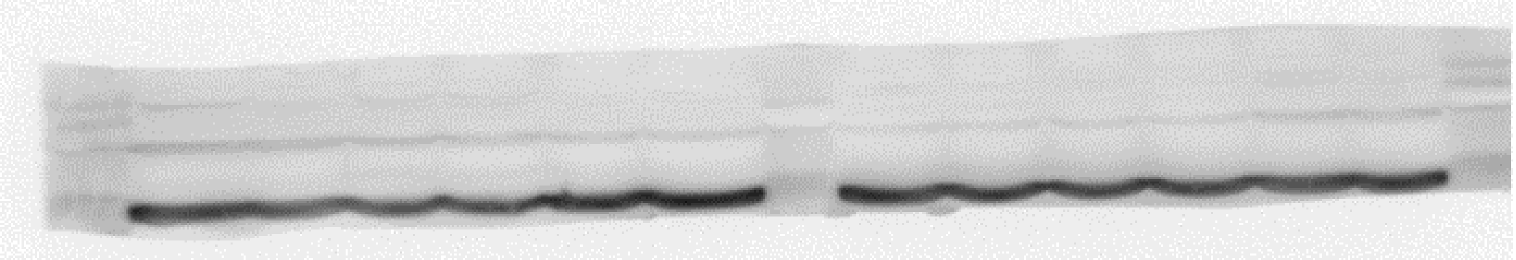

HEB HEB-H U87-MG U87-MG-H LN229 LN229-H U251 U251-H 090116 090116-H 091214 091214-H

2nd

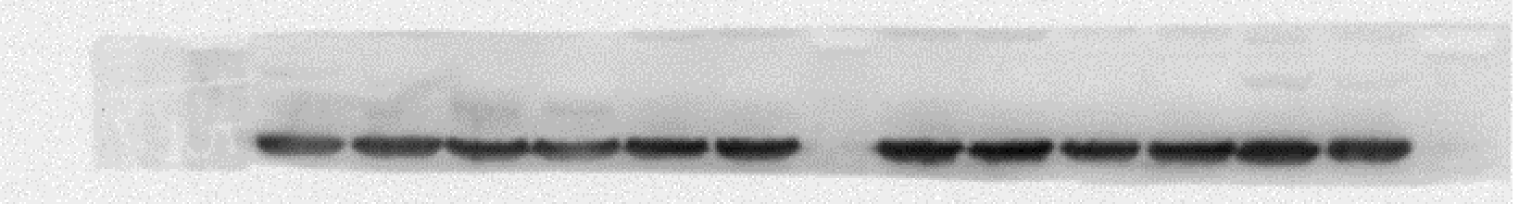

image in figure

U251 U251-H 090116 090116-H 091214 091214-H HEB HEB-H U87-MG U87-MG-H LN229 LN229-H

3rd

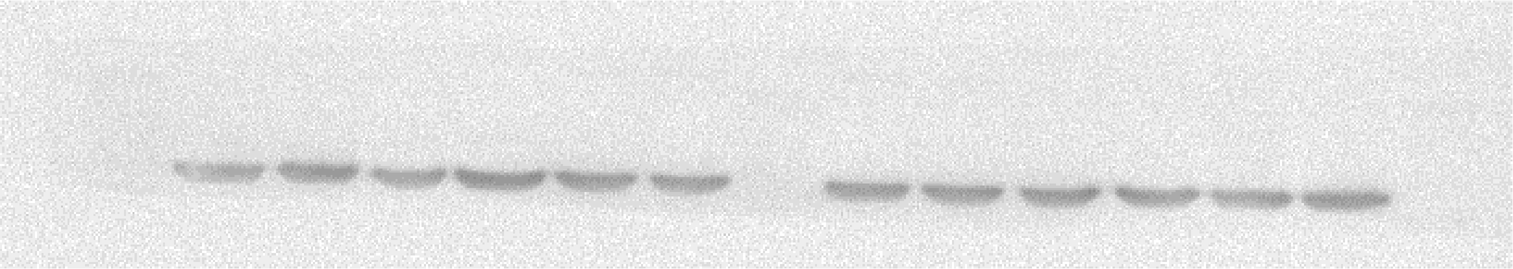

Supplement: Supplementary Figure 3 — The splicing data and the raw images of Figure 6B. Red arrows represent the locations of protein markers and green arrows represent the locations of splicing. [file Data_Sheet_1.pdf]
