# Supplementary material for: Targeting JUN, CEBPB, and HDAC3: A Novel Strategy to Overcome Drug Resistance in Hypoxic Glioblastoma
Source: Front Oncol. 2019 Feb 1;9:33. doi: 10.3389/fonc.2019.00033 (PMC6367651; doi:10.3389/fonc.2019.00033)
Supplement: Supplementary Figure 4 — Repeated experiments of Figure 6B without splicing. The protein expression levels of JUN, CEBPB and HDAC3 in HEB, U87-MG, LN229, 091116, and 091214 cells following incubation of the cells under 21% O2 and 1% O2 conditions (H) for 48 h. [file Data_Sheet_2.pdf]

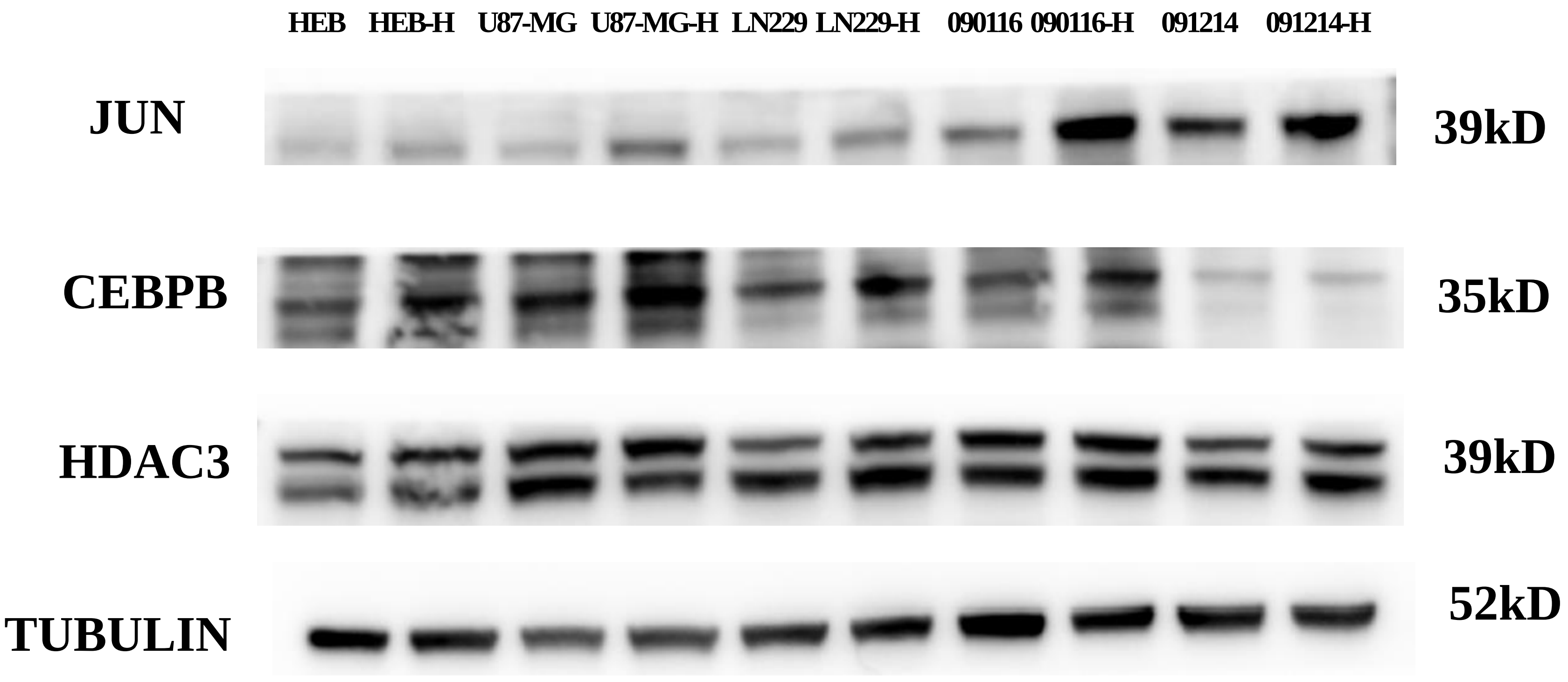
HEB
HEB-H
U87-MG
U87-MG-H
LN229
LN229-H
090116
090116-H
091214
091214-H
JUN
39kD
CEBPB
35kD
HDAC3
39kD
TUBULIN
52kD

anti-JUN

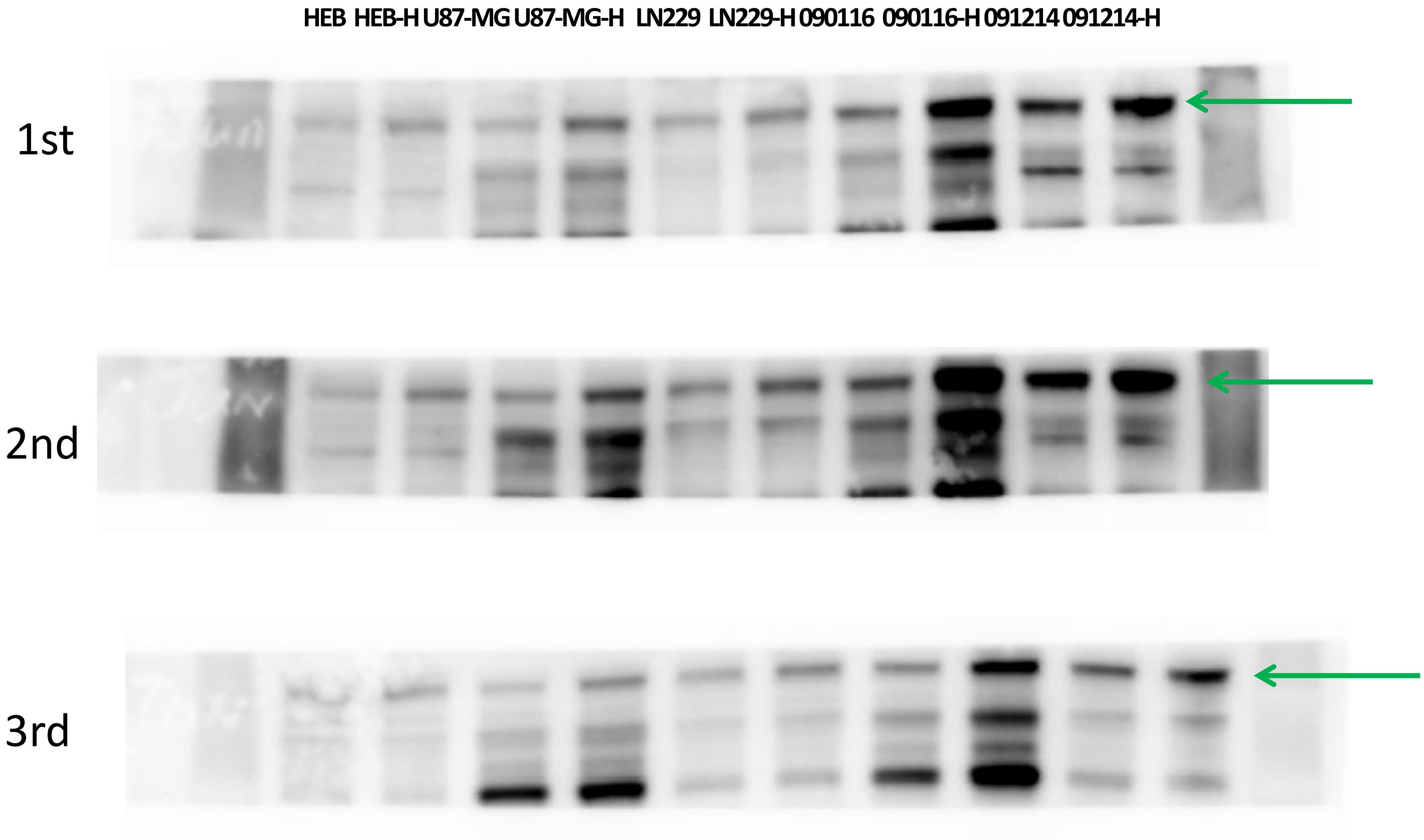
HEB
HEB-H
U87-MG
U87-MG-H
LN229
LN229-H
090116
090116-H
091214
091214-H
1st
2nd
3rd

anti-CEBPB

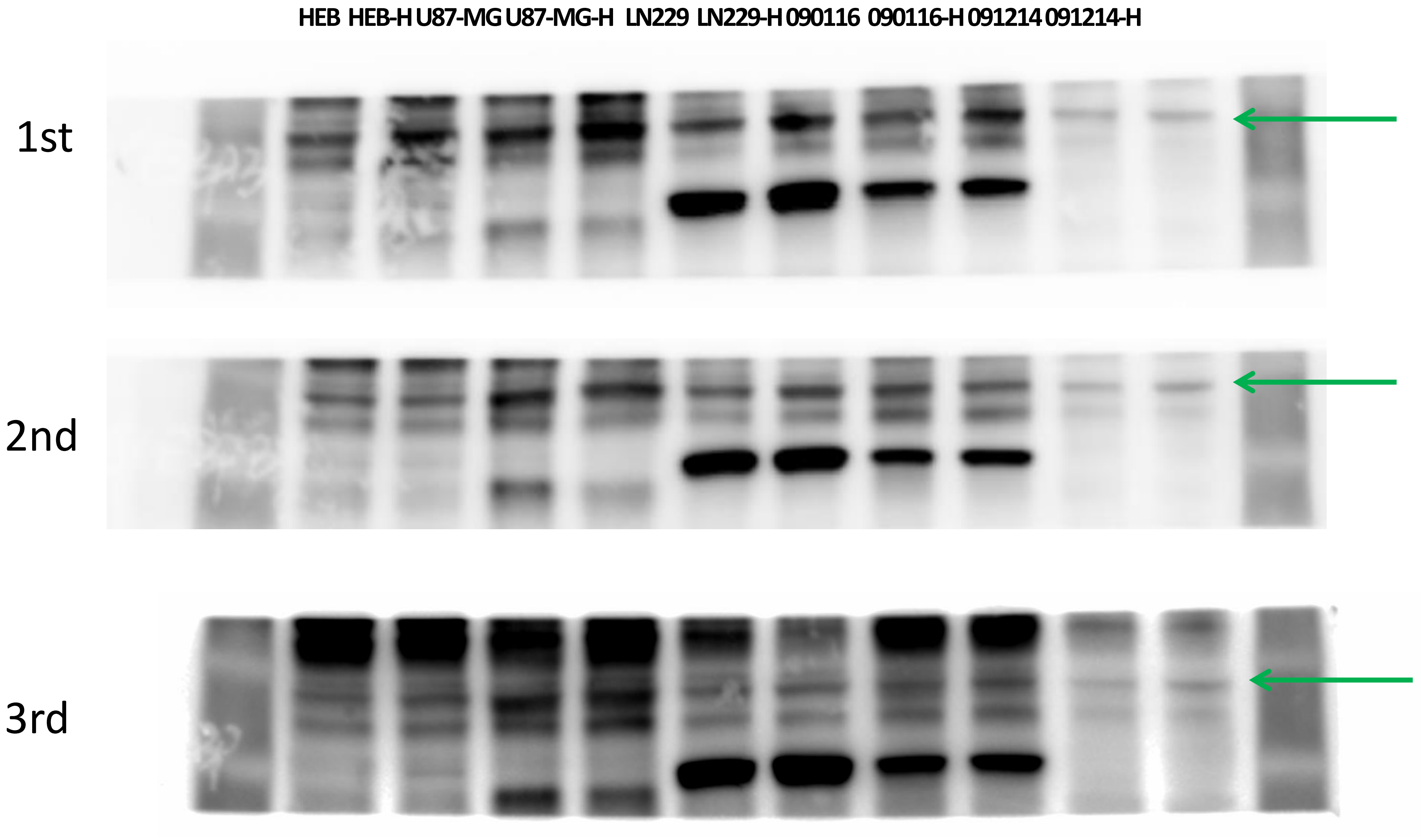
HEB HEB-H U87-MG U87-MG-H LN229 LN229-H 090116 090116-H 091214 091214-H
1st
2nd
3rd

anti-HDAC3

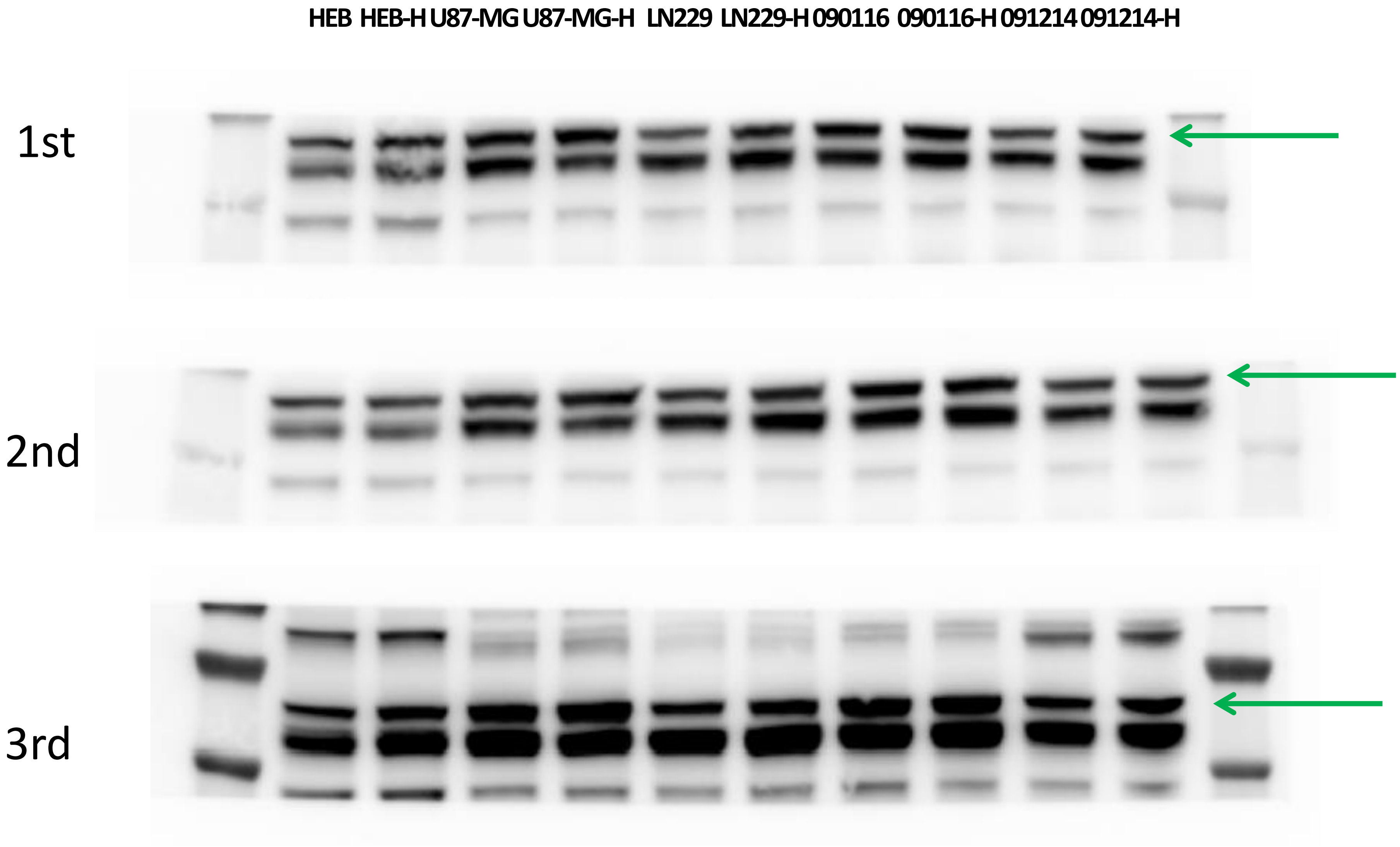

HEB HEB-H U87-MG U87-MG-H LN229 LN229-H 090116 090116-H 091214 091214-H
1st
2nd
3rd

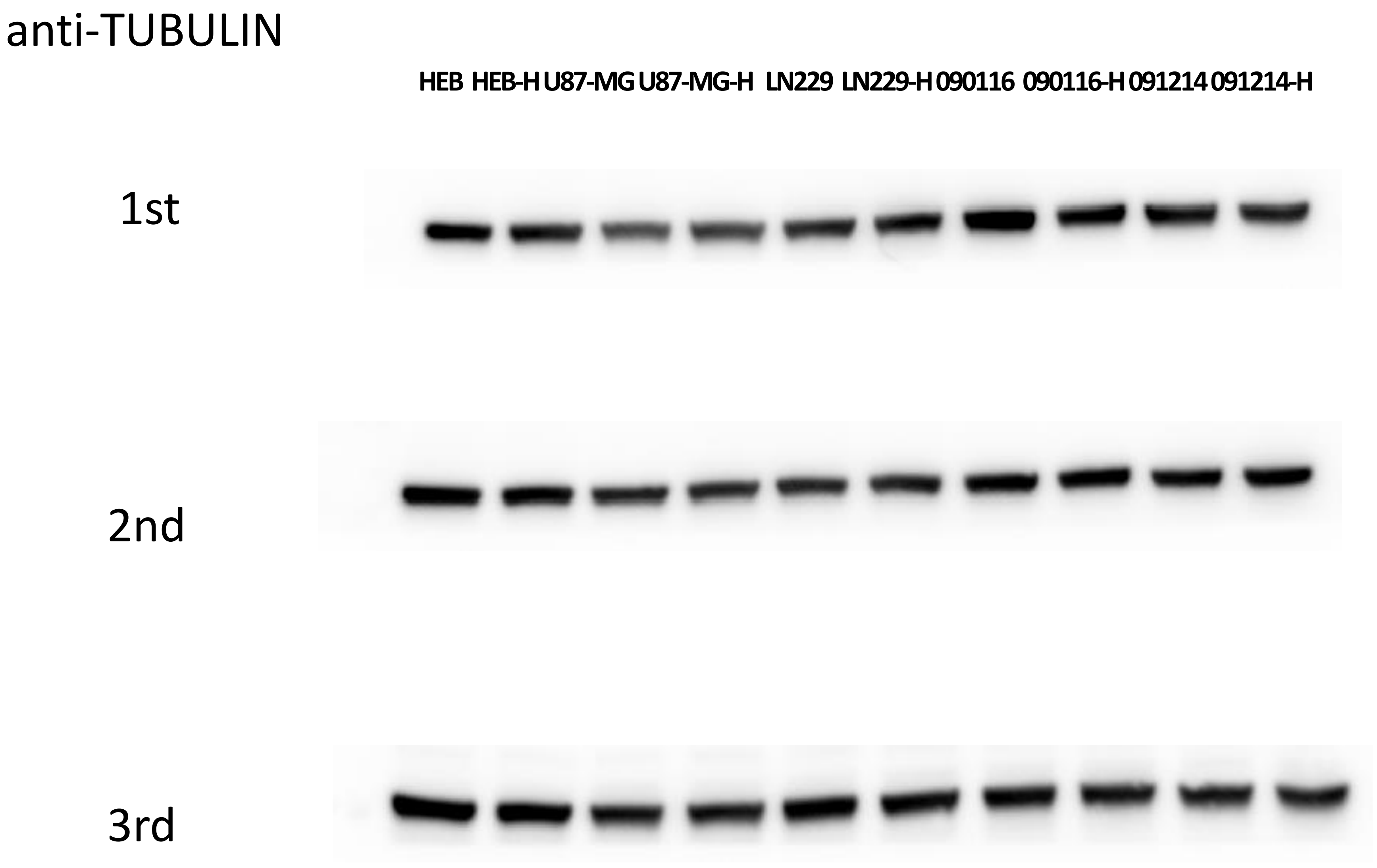
anti-TUBULIN
HEB
HEB-H
U87-MG
U87-MG-H
LN229
LN229-H
090116
090116-H
091214
091214-H
1st
2nd
3rd
