# Supplementary material for: Targeting JUN, CEBPB, and HDAC3: A Novel Strategy to Overcome Drug Resistance in Hypoxic Glioblastoma
Source: Front Oncol. 2019 Feb 1;9:33. doi: 10.3389/fonc.2019.00033 (PMC6367651; doi:10.3389/fonc.2019.00033)
Supplement: Supplementary Table 1 — Sequences of siRNA for knockdown of JUN, CEBPB and HDAC3. [file Table_1.docx]

Supplementary Table 1. Sequences of siRNA for knockdown of JUN, CEBPB and HDAC3

| siRNA name | Forward sequences(5’-3’) | Reverse sequences(3’-5’) |
| --- | --- | --- |
| JUN-homo-1552 | ACGCAAACCUCAGCAACUUTT | AAGUUGCUGAGGUUUGCGUTT |
| JUN-homo-1949 | GGAACAGGUGGCACAGCUUTT | AAGCUGUGCCACCUGUUCCTT |
| JUN-homo-1113 | GGACCUUAUGGCUACAGUATT | UACUGUAGCCAUAAGGUCCTT |
| CEBPB-homo-1889 | CCCUGAGUAAUCGCUUAAATT | UUUAAGCGAUUACUCAGGGTT |
| CEBPB-homo-1703 | CCGUGGUGUUAUUUAAAGATT | UCUUUAAAUAACACCACGGTT |
| CEBPB-homo-505 | CUGCCUUUAAAUCCAUGGATT | UCCAUGGAUUUAAAGGCAGTT |
| HDAC3-homo-311 | CCAAGAGUCUUAAUGCCUUTT | AAGGCAUUAAGACUCUUGGTT |
| HDAC3-homo-465 | GCACCAUGCCAAGAAGUUUTT | AAACUUCUUGGCAUGGUGCTT |
| HDAC3-homo-792 | GGUAGUGGACUUCUACCAATT | UUGGUAGAAGUCCACUACCTT |
